# Molecular assemblies and pharmacology of cerebellar GABA_A_ receptors

**DOI:** 10.1073/pnas.2524504123

**Published:** 2026-02-06

**Authors:** Chang Sun, Jennifer N. Jahncke, Kevin M. Wright, Eric Gouaux

**Affiliations:** ^a^Vollum Institute, Oregon Health and Science University, Portland, OR 97239; ^b^HHMI, Oregon Health and Science University, Portland, OR 97239

**Keywords:** cerebellum, ligand-gated ion channel, cryo-EM, heteromeric receptor assembly, pyrazoloquinolinone

## Abstract

GABA_A_ receptors (GABA_A_Rs) mediate fast inhibitory neurotransmission in the brain and are assembled from 19 subunit isoforms into multiple pentameric assemblies. Although α1-containing GABA_A_Rs are broadly expressed and are pharmacologically important, the molecular diversity of native α1-based assemblies in specific brain regions remains incompletely understood. Here, we use immunofluorescence, mass spectrometry, and cryogenic electron microscopy (cryo-EM) to characterize the spatial distribution, subunit composition, and structural architecture of native α1-containing GABA_A_Rs in the rat cerebellum. Confocal microscopy reveals robust colocalization of α1 and γ2 subunits across cerebellar layers, including prominent labeling at glomerular synapses. Biochemical purification and proteomic analysis identify a range of α, β, and γ subunits, along with abundant α6 and δ subunits. Using cryo-EM and automated subunit identification, we resolve eight α1-containing receptor assemblies, including the first structure of α6-containing receptors. We further determine the binding mode of the α6-selective pyrazoloquinolinone modulator PZ-II-029 at the α^+^/γ^–^ interface, showing ligand-induced expansion of the entire extracellular domain (ECD). Together, our study defines the structure and subunit composition of the α1-containing cerebellar GABA_A_Rs and elaborates the molecular interactions between native receptors and pyrazoloquinolinone, thereby laying the groundwork for brain region and subunit-specific pharmacology.

## Significance

Hetero-pentameric GABA_A_ receptors (GABA_A_Rs) are critical for inhibitory signaling in the brain, yet their molecular structure, subunit composition, and subunit arrangement remain incompletely defined, especially as a function of specific brain regions. Here, we reveal the molecular diversity of α1-containing receptors of the cerebellum using an integrated approach combining imaging, biochemistry, and cryo-EM. Our findings uncover cerebellar receptor assemblies composed of α1 and α6 subunits and define their nonrandom subunit arrangement within a conserved pentameric scaffold. This work advances our understanding of inhibitory receptor architecture and provides a structural framework for investigating region-specific receptor function and drug targeting.

Fast synaptic inhibition in the brain is primarily mediated by type A GABA receptors (GABA_A_Rs), ligand-gated, anion-selective ion channels that are activated upon binding the neurotransmitter GABA. By precisely regulating neuronal excitability, these receptors play a critical role in modulating sensory, motor, and cognitive functions (1, 2). GABA_A_Rs are important drug targets for treating stress, anxiety, insomnia, and seizure disorders, as well as for inducing general anesthesia (3, 4). Moreover, dysregulation of GABA_A_Rs is implicated in neurodevelopmental, neuropsychiatric, and neurodegenerative disorders, including autism spectrum disorder (5), schizophrenia (6), and Alzheimer’s disease (7). Despite their physiological and clinical importance, understanding the molecular mechanisms underlying GABA_A_R function in human health remains challenging due to the complex, heteromeric nature of these receptors, which are assembled from 19 different subunits (α1-6, β1-3, γ1-3, ρ1-3, π, δ, θ, ε). At present, our knowledge of native receptor assemblies and their functional diversity is limited.

Recent studies employing high-affinity antibodies targeting the broadly expressed α1 subunit have succeeded in the isolation of native GABA_A_Rs from mouse (8) and human (9) brain tissues. To maintain their structural integrity, these receptors were reconstituted into lipid-filled nanodiscs, enabling detailed investigation of native receptor diversity through complementary methodologies. Mass spectrometry-based proteomic analyses detected all six α, three β, three γ, and the δ subunits, suggesting a complex pool of receptors that coassemble with the α1 subunit (8, 9). Single-molecule photobleaching further revealed variability in α1 subunit stoichiometry, demonstrating that approximately half of the α1-containing receptors contain two α1 subunits, while the remainder possess one α1 subunit (8). High-resolution structures obtained via cryogenic electron microscopy (cryo-EM) elucidated diverse assembly configurations involving α1, α2, α3, β1, β2, β3, and γ2 subunits, including receptors featuring a β-β interface (9). Additionally, these high-resolution structures provide valuable insights into how native receptors interact with endogenous neurosteroids and exogenous drug molecules (8, 9).

Considering the regional expression patterns of GABA_A_R subunits within the brain, it is widely accepted that distinct brain regions harbor specific GABA_A_R assemblies. Expanding upon recent advances in structural biology studies of native GABA_A_Rs, we have now focused on the cerebellum—a region critical for fine motor coordination, emotional regulation, and social behaviors (10, 11). Because cerebellar GABA_A_Rs are less abundant than those in the cerebrum, we used rats instead of mice, taking advantage of approximately fivefold larger cerebellar mass of rats to facilitate biochemical and structural analyses. As a first step of this investigation, we imaged cerebellar slices using our α1-specific antibody to illuminate the spatial distribution of the α1-containing GABA_A_Rs. We then isolated cerebellar GABA_A_Rs using affinity purification and prepared them in lipid nanodiscs. With these native protein samples, we conducted protein composition analyses via mass spectrometry and elucidated their structures using cryo-EM.

## Results

### Spatial Distribution of α1-Containing GABA_A_Rs in the Cerebellum.

**Fig. 1.**
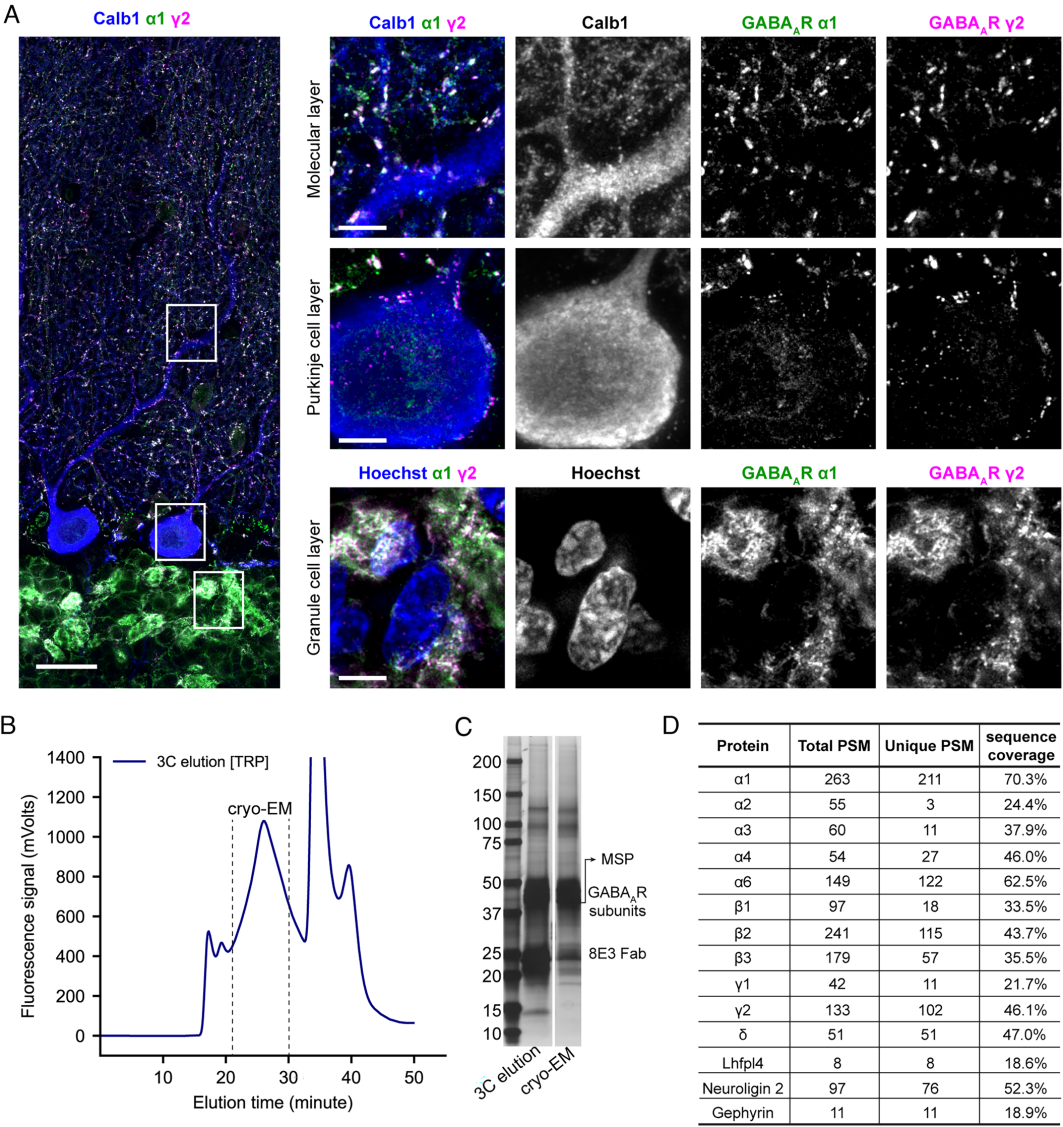
Localization and purification of α1-containing cerebellar GABA_A_Rs. (*A*) Immunofluorescence imaging of rat cerebellar cortex showing the spatial distribution of the GABA_A_ receptor (GABA_A_R) α1 and γ2 subunits. The α1 subunit was labeled with the monoclonal 8E3 antibody, and the γ2 subunit with a commercial polyclonal antibody. Calbindin staining highlights Purkinje cell somas and dendritic arborization, while Hoechst marks cell nuclei. The *Left* panel presents an overview composite image of Calbindin (blue), α1 (green), and γ2 (magenta). (Scale bar, 25 μm.) The *Right* panels show higher magnification views of boxed regions from the overview image, corresponding to different layers of the cerebellar cortex. (Scale bars, 5 μm.) (*B*) Tryptophan fluorescence-detection size exclusion chromatography (FSEC) traces of the cerebellar receptor preparation. Fractions indicated by the dotted lines in the 3C protease elution trace were pooled for cryo-EM sample preparation. (*C*) Silver staining SDS-PAGE analysis of the 3C protease elution and the final cryo-EM sample. (*D*) GABA_A_R subunits and auxiliary proteins identified from the cryo-EM sample using mass spectrometry. Both the total peptide spectrum match (PSM) and the unique PSM counts are listed.

To investigate the spatial distribution of α1-containing GABA_A_Rs in the cerebellum, we conducted confocal immunofluorescence imaging of rat cerebellar slices. Specifically, we utilized our in-house developed monoclonal antibody 8E3 (8, 12) to define the location of α1-containing receptors, a polyclonal γ2-specific antibody to identify synaptic receptors, a monoclonal calbindin 1 antibody to mark Purkinje neurons, and Hoechst staining to label nuclei (Fig. 1*A*). Within the molecular and Purkinje cell layers, robust colocalization of the α1 and γ2 subunits is observed. Individual puncta are detected along dendritic processes and the somas of Purkinje cells, indicative of inhibitory synapses from stellate and basket cells (13) (Fig. 1*A*). In contrast, the granule cell layer exhibits a more extensive labeling of GABA_A_R α1 and γ2 subunits, forming a characteristic pattern resembling glomerular structures (14, 15). This strong signal in the granule cell layer is consistent with the extensive GABAergic innervation of Golgi cells onto cerebellar granule cells (16, 17). To quantify the degree of colocalization between α1 and γ2 subunits, we analyzed confocal image stacks using threshold-based object detection in Imaris (*SI Appendix*, Fig. S1). Across the entire cerebellar cortex, more than 85% of α1 and γ2 fluorescence puncta overlap, with the highest colocalization observed in the granule cell layer. We further computed Pearson’s correlation coefficients, which are independent of thresholding, to further confirm the strong spatial correlation between the two subunits. The coefficients are approximately 0.9 in the granule cell layer, 0.7 in the molecular layer, and 0.6 in the Purkinje cell layer. In summary, the distinctive layer-specific distribution patterns of α1-containing GABA_A_Rs revealed by our imaging are consistent with previous immunohistochemistry studies (18, 19), but offer markedly enhanced spatial resolution. These results further support the use of 8E3 antibody as a valuable tool to label α1-containing GABA_A_Rs in situ.

### Biochemical Characterization of α1-Containing Cerebellar GABA_A_Rs.

Upon validation of the 8E3 antibody for labeling of α1-containing GABA_A_Rs in the cerebellum, we isolated cerebellar receptors in lipid-filled nanodiscs using a recombinantly expressed 8E3 Fab. Because of the small amounts of receptor, fluorescence-detection size exclusion chromatography (20) (FSEC) is used to monitor the separation of excess Fab from the receptor-Fab complexes (Fig. 1*B*). The peak fractions are pooled, concentrated to ~0.05 mg/mL (with an OD_280 nm_ = 0.08), and used for mass spectrometry, cryo-EM, and radioligand binding assays. Silver staining of SDS-PAGE gel (Fig. 1*C*) confirms sample purity, with distinct bands for membrane scaffold protein, Fab, and native receptor subunits.

With the native receptor samples in hand, we performed mass spectrometry analysis to elucidate the presence of specific receptor subunits as well as auxiliary or interacting proteins. We identified 11 GABA_A_R subunits and multiple auxiliary proteins, including lhfpl4, neuroligin 2, and gephyrin (Fig. 1*D*). Among the α subunits, α1 is the most highly represented, with 263 total peptide-spectrum matches (PSMs) and a sequence coverage of 70.3%. Notably, the α5 subunit, which is primarily associated with hippocampal function (21), is not detected in the cerebellar preparation. Other α subunits, including α2, α3, and α4, are detected at low abundance, whereas α6 exhibits a much higher PSM count. The relative abundance of α isoforms from mass spectrometry analysis is consistent with previous immunoprecipitation studies (22, 23). All β subunits are detected, with the β2 subunit displaying the highest peptide counts. Furthermore, γ2 and δ subunits are abundantly detected, suggesting the presence of both synaptic and extrasynaptic receptors within the α1-containing receptor preparation.

To corroborate the proteomics findings, we assessed the efficiency of membrane solubilization and affinity capturing using western blot analysis for all six α subunits (*SI Appendix*, Fig. S2). Assuming complete solubilization of receptors by SDS sample buffer, immunoblot quantification indicates that approximately 50% of the α1-containing receptors are solubilized from membranes, and of this fraction, ~90% are captured by the affinity resin. In contrast, α2 and α3 subunits are not detected, suggesting either lower abundance in cerebellar tissue or limited sensitivity of the primary antibodies used. While the α4, α5, and α6 subunits are all efficiently solubilized via detergent treatment, notable differences emerge in their capture efficiency by the affinity resin: Only the α6 subunit achieves appreciable capture (approximately 15%), whereas the α4 and α5 subunits show no measurable capture. Overall, these results demonstrate that our receptor purification based on 8E3 Fab recovered about half of the α1 subunit from tissue and further support the presence of α1/α6 receptor complexes in the cerebellum.

### Molecular Assemblies of Cerebellar GABA_A_R.

To elucidate the structures of the receptors, we prepared cryo-EM grids covered with a 2-nm carbon supporting film and collected two large datasets with different ligand conditions—either with GABA alone or with GABA plus PZ-II-029, an α6-selective positive allosteric modulator (PAM) of GABA_A_Rs (24, 25) (*SI Appendix*, Table S1). Notably, we used a combination of tilted and un-tilted stage data collection for the PZ-II-029/GABA dataset to mitigate the preferred top–down particle orientation. In data analysis for both datasets (*SI Appendix*, Figs. S3–S5), more than 2 million GABA_A_R particles exhibiting salient receptor features in 2D class averages are obtained following a simple cryo-EM cleanup workflow (*SI Appendix*, Figs. S3*A* and S5*A*). The large number of particles enables further data analysis to separate and identify distinct receptor assemblies.

Building on previous cryo-EM investigations (8, 9), we separated these GABA_A_R particles into different assemblies using a tandem 3D classification approach. In the first stage, particles are sorted based on the number of bound Fab molecules. Different filtering resolution parameters (3, 5, and 7 Å) consistently yield two-Fab, one-Fab, and ambiguous classes (*SI Appendix*, Fig. S3*B*). Notably, the assignment of particles is highly reproducible for the two-Fab classes (>85% overlap across independent runs), whereas reproducibility is substantially lower for the one-Fab classes (30 to 40%; *SI Appendix*, Fig. S3 *C* and *D*). This disparity underscores the utility of antibody labeling in enhancing 3D classification (26): The presence of two bound Fabs provides stronger structural features than one Fab, resulting in more robust particle alignment and classification. The lower reproducibility observed for the one-Fab group is primarily attributable to particles that defied robust classification, possibly because they are damaged or misaligned receptors. Upon exclusion of these ambiguous particles, classification reproducibility markedly improved for both one-Fab and two-Fab groups. Therefore, only clearly defined one-Fab and two-Fab classes were carried forward for subsequent analyses.

In the second stage, we independently classified the one-Fab and two-Fab populations based on glycosylation and side chain differences among GABA_A_R subunits. Because these differences are subtle relative to Fab occupancy, this classification step is inherently more challenging. Nevertheless, all resulting classes are refined to high resolution (2.7 to 3.4 Å, *SI Appendix*, Figs. S4 and S5). While several classes exhibit insufficient map quality in one or more positions, precluding confident subunit assignment, others yield maps with clear structural features to identify subunits at all five positions to define receptor assembly (*SI Appendix*, Figs. S4 and S5).

**Fig. 2.**
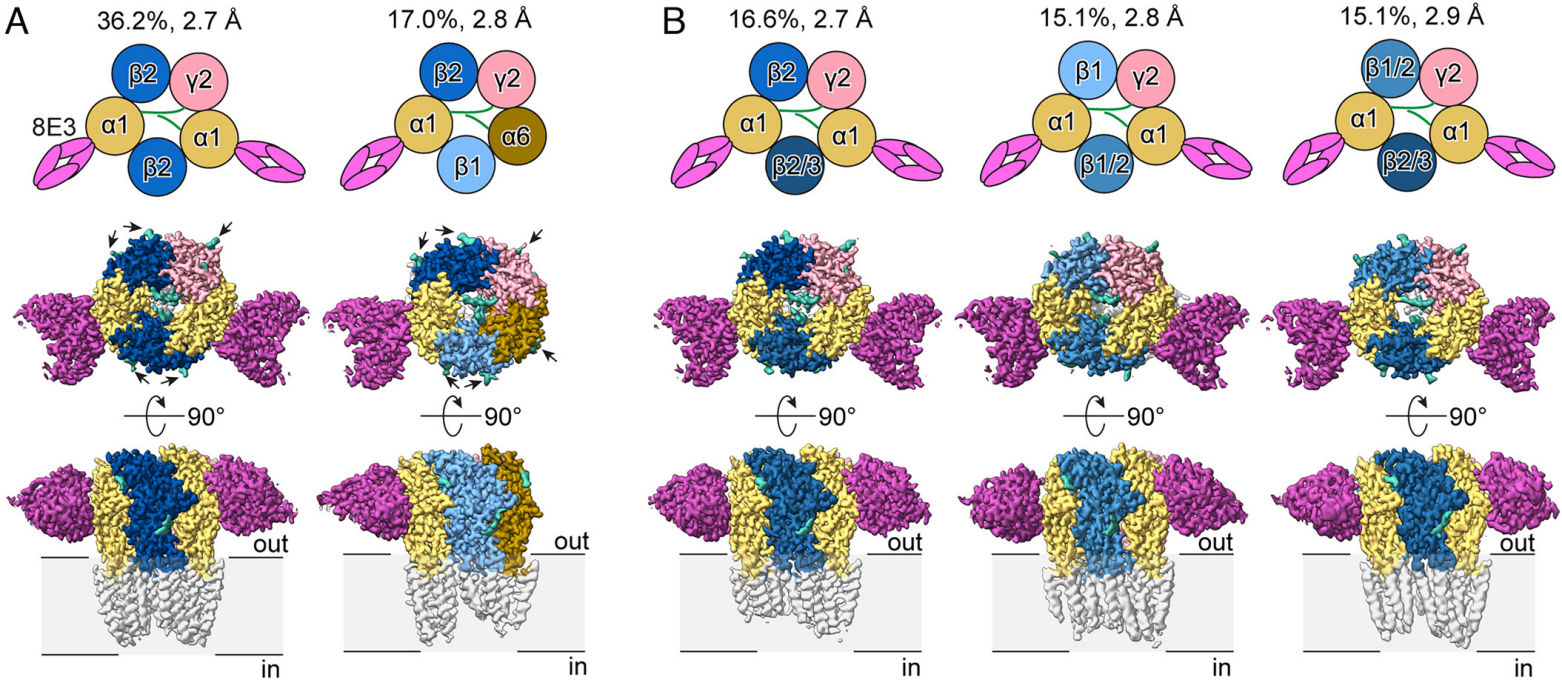
α1-containing cerebellar GABA_A_Rs show distinct α and β subunit combinations. Cryo-EM data analysis of the PZ-II-029/GABA dataset identifies five distinct receptor assemblies. (*A*) Two predominant assemblies with well-defined subunit identity, β2-α1-β2-α1-γ2 (viewed from the extracellular space, subunits counted counter-clockwise) and β2-α1-β1-α6-γ2. (*B*) Additional assemblies showing ambiguous density at one or both β subunit positions, corresponding to β2-α1-β2/3-α1-γ2, β1-α1-β1/2-α1-γ2, β1/2-α1-β2/3-α1-γ2. When two subunits are listed at one position, the first one denotes the predominant identity. Percentages of each receptor assembly are calculated based on the number of final particles used for the cryo-EM reconstruction (*SI Appendix*, Fig. S5). The extracellular domain (ECD) is colored based on the subunit identity. All *N*-glycosylation is colored in teal, and representative glycosylation at the ECD periphery is labeled with an arrow.

From the PZ-II-029/GABA dataset, five receptor assemblies are resolved at near-atomic resolution (2.7 to 2.9 Å) (Fig. 2 and *SI Appendix*, Fig. S6). The most prevalent assembly (36.2%) is comprised of β2-α1-β2-α1-γ2 subunits. Notably, a distinct population comprising β2-α1-β1-α6-γ2 is identified—representing a structural observation of this α6-containing assembly. Additional assemblies containing two α1 subunits include β2-α1-β2/3-α1-γ2, β1-α1-β1/2-α1-γ2, and β1/2-α1-β2/3-α1-γ2 receptors. Similarly, five assemblies are identified from the GABA dataset (*SI Appendix*, Fig. S4), including β1/2-α1-β2/1-α1-γ2, β1-α1-β2-α1-γ2, β2-α1-β2/3-α1-γ2, β2-α1-β2-α1-γ2, and another α6-containing assembly β2-α1-β1/3-α6-γ2. While mass spectrometry data detect all α, β, and γ subunits except for α5 and γ3, cryo-EM analysis reveals receptor assemblies from a more restricted subunit pool in cerebellum, namely α1/α6, β1/β2/β3, and γ2 subunits. This discrepancy is simply grounded in the higher detection sensitivity of mass spectrometry relative to cryo-EM. Taken together, cryo-EM analysis reveals a total of eight distinct receptor assemblies, all conforming to a stereotypical β-α-β-α-γ subunit arrangement.

Atomic models were constructed for the defined receptor assemblies. Due to limited resolution in the transmembrane domain (TMD) (*SI Appendix*, Fig. S6), model building was restricted to the extracellular domain (ECD) which harbors subunit-specific *N*-glycosylation sites and the binding pockets for the neurotransmitter GABA. In addition, distinct receptor assemblies were modeled only once per dataset. For instance, both β1/2-α1-β2/1-α1-γ2 and β1-α1-β2-α1-γ2 assemblies from the GABA dataset were represented by model β1-α1-β2-α1-γ2, and only the latter cryo-EM reconstruction was modeled. Eventually, three models from the GABA dataset and four from the PZ-II-029/GABA dataset were built and refined (*SI Appendix*, Fig. S6 and Table S2). The high resolution at the ECD (<3 Å) enables confident determination of side chain orientation (*SI Appendix*, Fig. S6). Indeed, manual inspection of the cryo-EM density with refined atomic models agrees with the subunit assignment by ModelAngelo (*SI Appendix*, Fig. S7), supporting the utility of an unsupervised computational approach for subunit identification.

### Architecture of the β2-α1-β1-α6-γ2 Receptor.

The GABA_A_R α6 subunit is almost exclusively expressed at the cerebellar granule cells (27) and contributes to distinct receptor properties compared to the more broadly expressed α1 subunit. Specifically, α6-containing receptors exhibit higher GABA sensitivity, shorter mean channel openings, and reduced desensitization (28, 29). Despite its crucial role in cerebellar function, little is known about the structure of α6-containing receptors and the pool of its native receptor assembly. Here, using the α1-specific antibody, we successfully purified receptors composed of α1 and α6 subunits from rat cerebellum and solved their cryo-EM structure from two different datasets. These structures support the existence of receptors with α1 and α6 subunits (6, 22) yet also shed light into their molecular architecture and pharmacology.

**Fig. 3.**
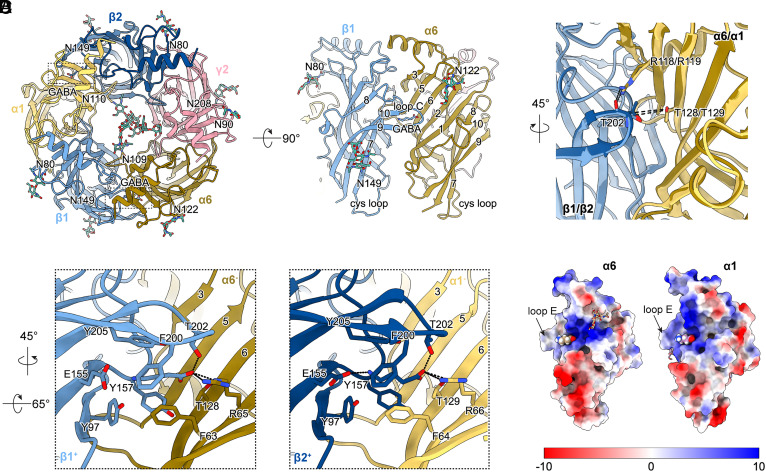
Cryo-EM structure of the β2-α1-β1-α6-γ2 cerebellar GABA_A_R in complex with GABA. (*A*) *Top-Down* and *Side* views of the ECD of the β2-α1-β1-α6-γ2 receptor. Key *N*-glycosylation is shown in stick representation, and prominent secondary structure elements are labeled. (*B*) Superimposed atomic models of β2^+^/α1^−^ and β1^+^/α6^−^ interfaces reveal a closed loop C conformation in both cases. The Cα distance between the threonine pair is 9.3 Å in both interfaces, same as the distance from previous native receptor structure bound with GABA (PDB: 8FOI). (*C*) Close-up view of the GABA-binding pockets at β1/α6 (*Left*) and β2/α1 (*Right*) interfaces, illustrating conserved side-chain arrangements at both binding interfaces. (*D*) Comparison of the electrostatic surface potential between α6 and α1 subunits. The loop E (between β4 and β5 strands) at the complementary face is labeled as a structural landmark. The *N*-glycosylation is shown in stick representation while the bound GABA molecule is shown in sphere representation.

The ECD of β2-α1-β1-α6-γ2 receptor displays characteristic *N*-glycosylation at both the extracellular-facing receptor surface and within the lining of the extracellular vestibule, consistent with previous structures of α1βγ2 receptors (12, 30, 31) (Fig. 3*A*). As predicted from the protein sequence, the α6 subunit exhibits a well-defined *N*-linked glycosylation (N109) site inside the receptor vestibule, which likely limits the receptor assembly to no more than two α6 subunits. Unlike α1, the α6 subunit harbors an additional *N*-linked glycosylation site at the periphery of the ECD (N122), located on the short loop between β strands 5 and 6. Although this *N*-linked glycosylation site is positioned similarly to the N80 site of β subunits, it can be distinguished upon close inspection, even at an intermediate resolution of ~5 Å. While only two *N*-acetylglucosamine molecules were modeled based on cryo-EM maps, the actual glycan chain likely extends further and may interact with the GABA-binding loop C of an adjacent β subunit.

Given that the α6 subunit has different residues from α1 throughout the ECD (~67% sequence identity; *SI Appendix*, Fig. S8 *A* and *B*), we compared the structures between α1 and α6 and investigated the structural consequence of incorporating both subunits into the same receptor assembly. Despite the amino acid sequence differences, the ECD backbone of α6 closely resembles that of α1, with a Cα RMSD of 0.67 Å and almost all backbone differences exclusively observed at loop regions (*SI Appendix*, Fig. S8*C*). Overall, the ECD of the β2-α1-β1-α6-γ2 receptor exhibits near 5-fold symmetry (Fig. 3*A*), as further supported by subunit centers-of-mass analysis (*SI Appendix*, Fig. S9*A*). Focusing on the GABA binding sites, we find both β2^+^/α1^–^ and β1^+^/α6^–^ interfaces displayed a similarly closed loop C conformation, featuring a hydrogen bond from the α1-R119/α6-R118 side chain to β2-Τ202/β1-Τ202 backbone carbonyl (Fig. 3*B*). Close-up views of the ligand-binding sites reveal nearly identical side-chain arrangements involving Y97, E155, Y157, F200, T202, and Y205 from the principal face of β subunits, and R66/R65 and F64/F63 from the complementary face of α subunits. While the hydrogen bonds involved in the carboxylate group of GABA are preserved in the β1^+^/α6^–^ pocket, the hydrogen bond between the amine group of GABA and the E155 side chain is absent (Fig. 3 *C* and *D*), reflecting subtle structural differences between these two pockets, which may translate into different ligand binding affinities. Furthermore, despite backbone similarity, the electrostatic surface potentials of α6 and α1 diverge markedly, particularly around the GABA binding pocket, which could influence GABA entry/exit kinetics (Fig. 3*E*).

In summary, our β2-α1-β1-α6-γ2 receptor structures demonstrate that α6 can structurally substitute for α1 without altering the overall pentameric architecture or ligand-binding geometry. These findings suggest a surprisingly high degree of structural compatibility between α1 and α6 subunits and demonstrate the presence and physiological relevance of mixed α1/α6 GABA_A_R assemblies in the cerebellum.

### Structural Comparison of α1-Containing GABA_A_R Assemblies.

To evaluate the structural impact of subunit variation in cerebellar GABA_A_R assemblies, we performed pairwise comparisons of representative α1-containing receptors resolved from the GABA dataset. These include three distinct pentameric arrangements β2-α1-β2-α1-γ2, β1-α1-β2-α1-γ2, and β2-α1-β1-α6-γ2. Despite differences in α and β subunit identity, the overall architecture of the ECD is highly similar across assemblies. Full ECD alignment between β2-α1-β2-α1-γ2 and β1-α1-β2-α1-γ2 yields a global Cα RMSD of 0.57 Å (*SI Appendix*, Fig. S10*A*), indicating minimal perturbation of the pentameric scaffold upon β subunit substitution. Likewise, comparison between β1-α1-β2-α1-γ2 and the α6-containing assembly β2-α1-β1-α6-γ2 results in a global RMSD of 0.58 Å (*SI Appendix*, Fig. S10*B*), highlighting the structural compatibility of α6 with canonical α1-based configurations.

Analysis at the individual subunit level reveals that structural divergence is influenced by both subunit isoform and positional context within the pentamer (*SI Appendix*, Fig. S10). The largest conformational differences are observed between α1 and α6 subunits (RMSD 0.63 to 0.76 Å), whereas the differences between β1 and β2 subunits are more modest (RMSD 0.24 to 0.52 Å). Notably, RMSD values between β1 and β2 subunits occupying the same position are often smaller than those observed for a given β subunit at different positions, suggesting that local environment and intersubunit interactions may play a more prominent role in shaping subunit conformation than sequence differences between isoforms. Collectively, these structural comparisons underscore the architectural robustness of the GABA_A_R, capable of incorporating a range of α and β subunit combinations.

### Binding of PZ-II-029 to Cerebellar GABA_A_Rs.

**Fig. 4.**
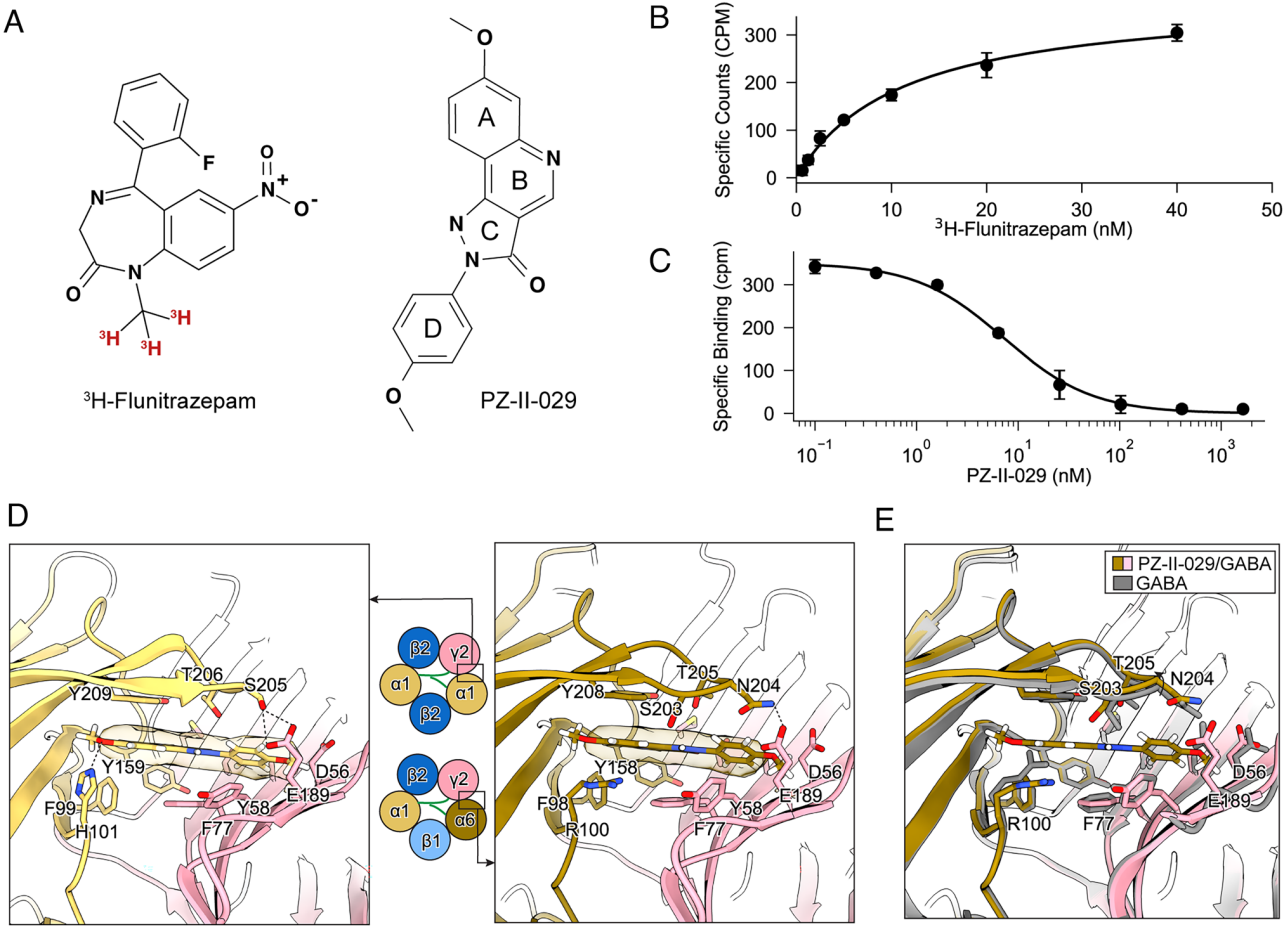
Molecular interactions between cerebellar GABA_A_Rs and pyrazoloquinolinone ligand PZ-II-029. (*A*) Chemical structures of the radioligand [^3^H]-flunitrazepam and the nonradioactive ligand PZ-II-029. The letters A, B, C, and D refer to the different rings in the pyrazoloquinolinone scaffold. (*B*) Saturation binding curve of [^3^H]-flunitrazepam to cerebellar GABA_A_Rs, showing specific binding in counts per minute (CPM) as a function of ligand concentration. (*C*) Competitive binding assay showing displacement of [^3^H]-flunitrazepam by PZ-II-029, plotted as specific binding versus PZ-II-029 concentration. (*D*) PZ-II-029 binding to the α1/γ2 pocket (*Left*) and the α6/γ2 pocket (*Right*). The cryo-EM density of the ligand is shown as a transparent surface. (*E*) Structural overlay of the PZ-II-029/GABA-bound (yellow/pink) and GABA-only (gray) structures reveals conformational changes in the binding site, including sidechain movements and the slight displacement of loop C to accommodate the ligand.

PZ-II-029 (Fig. 4*A*) is a pyrazoloquinolinone-based compound originally identified as a highly selective PAM of the α6β3γ2 subtype of GABA_A_R (25) and holds potential for treating a range of neurological diseases including schizophrenia and autism spectrum disorder (6). Detailed pharmacological studies showed PZ-II-029 can bind to both the canonical α^+^/γ^−^ benzodiazepine pocket as well as to the α6^+^/β^−^ interface (25). However, the molecular details of the interactions between GABA_A_R and PZ-II-029, or pyrazoloquinolinones in general, at both sites have remained elusive. Given the high expression of α6 subunit in cerebellar granule cells, which are the most numerous neurons in the brain, we reasoned our cerebellar receptor preparation would be an ideal sample to provide structural insights into PZ-II-029 binding.

We first carried out scintillation proximity assays (SPA) using ^3^H-flunitrazepam to probe the functionality of the benzodiazepine pocket of our protein sample. Flunitrazepam binds to the cerebellar receptor with an affinity of 11.1 ± 1.2 nM (Fig. 4*B*). Compared to whole-brain receptors (8), the cerebellar receptor exhibits a 2.5-fold higher Kd, suggesting a distinct population of benzodiazepine binding sites in the cerebellum. The K_i_ of PZ-II-029 is determined to be 4.2 ± 0.4 nM (Fig. 4*C*). These results confirm functional ligand binding in the cerebellar preparation and demonstrate the competitive binding of PZ-II-029 at the benzodiazepine site.

From the PZ-II-029/GABA dataset, all four modeled GABA_A_R assemblies are bound with PZ-II-029 at the benzodiazepine pocket (*SI Appendix*, Fig. S6 and Table S2). At the α1/γ2 interface, the density for PZ-II-029 is contiguous and appears as a “boomerang”, which is consistent with the compound’s predicted elongated and near-planar geometry. Structural modeling and refinement reveal a ~30° rotation of the ring D with respect to rings A, B, and C of PZ-II-029, with the ring D pointing toward the γ2 subunit (Fig. 4*D* and *SI Appendix*, Fig. S11). Within this pocket, PZ-II-029 engages in multiple π-stacking interactions with the surrounding residues, including F99, Y159, and Y209 from the principal face of α1 subunit and Y58 and F77 from the complementary face of the γ2 subunit. Furthermore, PZ-II-029 forms a hydrogen bond with α1-H101 using its methoxy oxygen on ring A. Our structure confirms a pyrazoloquinolinone binding model at the α1/γ2 benzodiazepine site proposed in a previous computational study (32). Interestingly, the hydrogen bond between S205 of the α1 loop C and D189 of the γ2 strand 9 is intact, effectively locking the bound PZ-II-029 in place.

PZ-II-029 is also observed to bind at the α6^+^/γ2^−^ pocket, engaging in largely similar molecular interactions. One key difference, however, is that the hydrogen bond with α1-H101 is substituted by a weaker π-cation interaction with α6-R100, which could explain the reported lower affinity of PZ-II-029 toward α6/γ2 pocket (25). This difference, however, does not readily explain the functional selectivity of PZ-II-029 toward α6-containing receptors, which was attributed mainly to the α6^+^/β^−^ interface in prior pharmacological studies (25). Compared to the corresponding GABA-only structures, rearrangements of side chains at the binding pocket are observed upon binding of PZ-II-029, most notably that of α6-R100, which shifts by 2.6 Å in order to not clash with the modulator (Fig. 4*E*). The loop C of the α6 subunit is pushed slightly outward, but the hydrogen bonds between the α6 loop C and γ2-E189 are preserved (Fig. 4*D*). Outside of the binding pocket, binding of PZ-II-029 leads to rearrangement of the ECD of all five subunits, rendering them in a more extended conformation along the channel pore axis (*SI Appendix*, Fig. S12). Overall, the incorporation of PZ-II-029 into the α^+^/γ^−^ pockets of GABA_A_R increases the intersubunit distances consistently—0.2 Å for γ-α, 0.2 to 0.5 Å for β-α, and 0.5 to 0.6 Å for γ-β (*SI Appendix*, Fig. S9). Similar effects were observed previously with a classical benzodiazepine for the γ-α distance, but not for the other intersubunit distances (33), suggesting that binding of PZ-II-029 to GABA_A_Rs has different structural consequences than benzodiazepines.

## Discussion

Since the initial biochemical purification of GABA_A_Rs four decades ago (34), its structural complexity—particularly with respect to subunit stoichiometry and arrangement—has garnered sustained research interest, as it underpins the receptor’s functional and pharmacological diversity (3, 4, 35). Although early biochemical and electrophysiological studies (36–40) laid a critical framework, cryo-EM has enabled direct visualization of receptor assemblies and associated ligand binding, substantially advancing our understanding of GABA_A_Rs (12, 30, 31, 33). Over the past three years, cryo-EM studies have made significant strides in resolving the structural diversity of GABA_A_Rs. In 2022, Sente et al. resolved multiple receptor structures formed from recombinant β3-containing GABA_A_R samples, revealing noncanonical subunit stoichiometries with functional implications (41). Building on this progress, we elucidated three major α1-containing GABA_A_R populations in the mouse brain, uncovering widespread native receptors containing two different α subunits (8). Most recently, Zhou et al. (2024) reported an impressive collection of twelve native assemblies from surgically removed human brain tissue, providing the most comprehensive structural atlas of native GABA_A_Rs to date (9).

Building on previous GABA_A_R structural studies, we applied cryo-EM to investigate the receptor assemblies in rat cerebella. Most notably, our analysis captures high-resolution structures of native α6-containing receptors. These assemblies, which have five distinct subunits arranged as β2-α1-β1-α6-γ2 or β2-α1-β1/3-α6-γ2, contain nonidentical α and β subunits but maintain a conserved architecture at the GABA-binding sites, underscoring the structural compatibility of α and β subunits in assembling pentameric receptors. These observations may suggest a preferential association of β1 and β3 subunits with the α6 subunit in the cerebellum, although definitive demonstration of this hypothesis requires further experiments. Our cryo-EM analysis is further corroborated by immunofluorescence imaging which suggests the colocalization of α1 and α6 subunits and proteomic data which confirm the copurification of these subunits. Although a previous report proposed an alternative α1/α6 arrangement in which the two α subunits swap positions within the pentamer (42), we do not observe such a configuration in either dataset. Whether this alternative architecture reflects a low-abundance or context-dependent receptor form remains an open question. In addition to receptor composition, our study provides structural insight into the pharmacological mechanism of PZ-II-029, a pyrazoloquinolinone compound that selectively modulates α6-containing receptors. We observe clear ligand density for PZ-II-029 at both the α1-γ2 and α6-γ2 interfaces, along with conformational rearrangements caused by the binding. These changes include an outward displacement of loop C and a coordinated expansion across all five ECDs, supporting a model in which binding of PZ-II-029 at the α-γ2 site expands the ECD and contributes to allosteric modulation.

Despite the advances of the research reported here, several limitations of the current study should be acknowledged. First, our immunofluorescence analysis focused on α1 and γ2 subunits; imaging using α6 was not performed due to the lack of a suitable α6-specific antibody. Second, although mass spectrometry identified a significant number of δ subunit peptides, we did not resolve any δ-containing receptor assemblies in the cryo-EM reconstructions. This may reflect low abundance, conformational heterogeneity, or the need for more sophisticated data analysis workflows. Direct labeling of the δ subunit may be necessary to uncover the arrangement and pharmacology of native δ-containing receptors in future studies. Third, the limited resolution in the TMD precluded detailed modeling of pore architecture or gating transitions. Resolving these features will require further optimization of receptor isolation and stabilization, grid preparation, and data acquisition.

Together, our results define the architecture and pharmacology of α1- and α6-containing GABA_A_Rs in the cerebellum, uncovering mixed α subunit assemblies and pyrazoloquinolinone ligand interaction modes that may inform region- and subtype-selective therapeutic strategies.

## Materials and Methods

### Recombinant α1-Specific Fab Production and Labeling.

The recombinant α1-specific mouse monoclonal antibody fragment (8E3 Fab), engineered with a 3C protease cleavage site, EGFP, and a twin Strep II affinity tag at the C-terminus of the heavy chain (8E3-GFP), was expressed, purified, and stored as previously described (8).

### Immunofluorescence Imaging of Cerebellar Slices.

Rats aged 3 mo were anesthetized with isoflurane, and the cerebella were rapidly excised. Cerebellar vermis was isolated and immersed in a fixative solution of 2% paraformaldehyde, 4.5% glyoxal, and 4% acetic acid in phosphate-buffered saline (PBS) pH 4.0 for 1 h at room temperature followed by storage in PBS with 0.002% sodium azide at 4 °C overnight. Sagittal sections were prepared on a vibratome (VT1200S, Leica Microsystems Inc, Buffalo Grove, IL) at a thickness of 70 µm. Sections were blocked at room temperature for 5 h in blocking solution containing 0.4% Triton X-100, 2% normal goat serum, 0.3 M glycine, and 0.002% sodium azide in PBS. The blocking solution was removed and replaced with primary antibodies in a carrier solution of 0.4% Triton X-100, 2% normal goat serum, and 0.002% sodium azide in PBS. The following primary antibodies were used: GABA_A_R α1 (8E3 mAb, mouse IgG2b, 8 ng/µL), GABA_A_R γ2 (Synaptic Systems, cat. no. 224 003, rabbit, 1:250), and Calb1 (Boster Bio, cat. no. M03047-2, chicken, 1:1,000). After incubation in primary antibody solution for 48 h at 4 °C, sections were washed 4 times in PBS for 15 min each at room temperature. Secondary antibodies in carrier solution were then applied for 48 h at 4 °C. The following secondary antibodies were used: goat anti-mouse IgG2b Alexa Fluor 488 (Invitrogen, cat. no. A-21141, 1:500), goat anti-rabbit Alexa Fluor 546 (Invitrogen, cat. no. A-11035, 1:500), goat anti-chicken Alexa Fluor 647 (Invitrogen, cat. no. A-21449, 1:500), and Hoechst 33342 (Invitrogen, cat. no. H3570, 1:10,000). Sections were washed 4 times in PBS for 15 min each at room temperature and then mounted on Superfrost Plus slides (Fisher, cat. no. 12-550-15) using Fluoromount-G mounting medium (SouthernBiotech, cat. no. 0100-01), cover slipped with No. 1 coverslips (Fisher, cat. no. 12-541-025) and sealed with clear nail polish. Imaging was performed on an upright Zeiss LSM 980 laser scanning confocal microscope with Airyscan 2 in Multiplex SR-4Y mode paired with a 63×/1.4 NA objective, allowing for a maximum resolution of 140 nm in the xy-plane and 450 nm in the z-direction. Z-stacks with 0.14 µm spacing spanning a depth of 15 µm were acquired at 1.2× zoom with tiles to capture cerebellar cortex and a portion of the granule cell layer. Confocal images were acquired from four individual rats, with three representative images per animal. Orthogonal maximum projection images were used for composite image display. Colocalization between the α1 and γ2 subunits was quantified in Imaris (Bitplane). For each image, colocalization analysis was performed over the entire field of view (“all”) as well as within manually defined regions of interest (ROIs), including the granule cell layer (GCL), Purkinje cell layer (PCL), and molecular layer (ML). Both the percentage of colocalized signal from one channel to another and the Pearson’s correlation coefficient between two channels are reported to quantitate the degree of colocalization.

### Cerebellar GABA_A_R Isolation and On-Column Nanodisc Reconstitution.

Thirty killed rats of mixed sexes (10 to 12 wk old) were donated by the John Williams lab (Vollum Institute). After dissection, the rat cerebella were isolated from the brain and washed twice with ice-cold 20 mM Tris and 150 mM NaCl (TBS) buffer. The tissue was resuspended in three volumes of ice-cold TBS buffer supplemented with two protease inhibitor cocktail tablets (Roche). Homogenization was performed using a loose-fit Potter-Elvehjem homogenizer with 20 full up-and-down strokes, followed by sonication at 20 W for 30 s at 50% amplitude using a mini probe. The homogenate was first centrifuged at 1,000×*g* for 10 min to pellet the nuclear fraction, which was discarded. The resulting supernatant and soft pellet were subjected to ultracentrifugation at 100,000×*g* for 20 min to isolate the membrane fraction. The membrane pellet was resuspended in TBS buffer containing 10% glycerol, at a final volume ~16 mL, aliquoted, and snap-frozen in liquid nitrogen.

The following protein preparation steps were all carried out at 4 °C. For solubilization, 8 mL of the membrane suspension was incubated with mixed maltose-neopentyl glycol (MNG) and cholesteryl hemisuccinate (CHS) (w/w ratio of 10:1) at a final concentration of 1.5% (w/v) for 1 h on a platform rocker. Before ultracentrifugation at 200,000×*g* for 1 h, 100 nM 8E3-GFP Fab was added to label native receptors. The supernatant was incubated with 2 mL streptactin XT 4 flow resin for 2 h to capture receptor-Fab complexes. Meanwhile, 40 nmol MSP2N2 (43) was incubated with dodecyl-β-D-maltopyranoside (DDM) solubilized bovine brain lipid extracts at a molar ratio of 1:80 in a final volume of 2 mL. The MSP/lipid mixture was then combined with the resin and incubated for 1.5 h. Subsequently, 200 mg of Bio-Beads were added, and incubation continued for 1 h. Following this, 100 µL of 15 µM 8E3-GFP Fab was added and Bio-Bead incubation was extended overnight. To evaluate the efficiency of the membrane solubilization and affinity capturing, western blot was carried out using a Li-Cor imager with commercial antibodies against α1-6 subunits (Alomone Labs, cat. no. AK-228_KIT) and IRDye® 800CW goat anti-rabbit IgG secondary antibody.

The following day, the resin was washed with approximately 25 column volumes of TBS to remove empty nanodiscs. Then, 4 mL of 3C protease at 0.1 mg/mL with 0.1 mM Tris (2-carboxyethyl) phosphine hydrochloride (TCEP) was added to the resin and incubated for 1 h. The resin was subsequently washed with 4 mL of TBS. The combined elution was concentrated using a 30-kDa centricon. The protein sample was then injected onto a Superose 6 increase 10/300 GL column connected to an HPLC for size exclusion chromatography (SEC). The GABA_A_R-Fab peak was monitored in the Tryptophan fluorescence channel. The peak was collected and concentrated to an OD_280 nm_ of ~0.08. Throughout the purification, 1 mM GABA was included in all the buffers from membrane solubilization to final SEC. The prepared sample was used for cryo-EM sample preparation immediately, or snap frozen in liquid nitrogen after adding 10% glycerol, for future biochemical analysis.

### Mass Spectrometry.

Mass spectrometry-based protein profiling of native receptors was performed as previously described (8). Briefly, the purified rat cerebellar GABA_A_R sample was dried, dissolved in 5% sodium dodecyl sulfate, 50 mM Triethylammonium bicarbonate buffer (TEAB; pH 8), reduced with Dithiothreitol (DTT) at 95 °C for 10 min, and alkylated with Iodoacetamide (IAA) in the dark for 30 min at room temperature followed by the addition of acidified 90% methanol and 100 mM TEAB (pH 7.55). The sample was loaded into an S-Trap micro column to capture the proteins and the column was washed with 90% methanol with 100 mM TEAB six times to remove SDS. The proteins were digested on column overnight with 3.2 µg of Trypsin dissolved in 40 μL 50 mM TEAB. The peptides were eluted with 50 mM TEAB, 50% acetonitrile, and 0.2% formic acid, then pooled, and dried. Each sample was dissolved in 20 µL of 5% formic acid and injected into the Thermo Fisher Orbitrap Eclipse mass spectrometer. Protein digests were separated using liquid chromatography with a Dionex RSLC UHPLC system, then delivered to an Orbitrap Eclipse (Thermo Fisher) using electrospray ionization with an EasySpray Source (Thermo Fisher). Xcalibur version 4.0 was used to control the system. Samples were applied at 5 µL min^−1^ to a PepMap C18 trap cartridge (Thermo) for 10 min, then switched onto a 75 µm × 250 mm RSLC C18 column with 2 µm particles (Thermo) using mobile phases water (A) and acetonitrile (B) containing 0.1% formic acid, 5 to 30% acetonitrile gradient over 70 min and a 300 nL min^−1^ flow rate. Survey mass spectra were acquired over m/z 350 to 1,400 at 60,000 resolution (m/z 200) in the Orbitrap and data-dependent acquisition selected the top 10 most abundant precursor ions for tandem mass spectrometry by higher energy collisional dissociation using an isolation width of 1.6 m/z, normalized collision energy of 30 and an Orbitrap resolution of 7500. Dynamic exclusion was set to 45 s, charge state for MS/MS +2 to +7, maximum ion time 50 ms, normalized AGC target of 100% in MS1 mode and 100% in MS2 mode. Data analysis was performed using Comet (v. 2016.01, rev. 3) against the *Rattus norvegicus* reference proteome (obtained in July 2024) from UniProt and concatenated sequence-reversed entries to estimate error thresholds and 179 common contaminant sequences and their reversed forms. Protein sequences for the light chain and heavy chain of 8E3 Fab were also included. Comet searches for all samples were performed with trypsin enzyme specificity with monoisotopic parent ion mass tolerance set to 1.25 Da and monoisotopic fragment ion mass tolerance set to 1.0005 Da. A static modification of +57.0211 Da was added to all cysteine residues and a variable modification of +15.9949 Da on methionine residues. A linear discriminant transformation was used to improve the identification sensitivity for the Comet analysis. Separate histograms were created for matches to forward sequences and for matches to reversed sequences for all peptides of seven amino acids or longer. The score histograms of reversed matches were used to estimate peptide false discovery rates (FDR) and set score thresholds for each peptide class. The overall protein FDR was 1.5%.

### Scintillation Proximation Assay.

Copper His-tag YSI SPA beads (PerkinElmer) were used to bind cerebellar GABA_A_Rs reconstituted in nanodiscs via the His-tag located at the N terminus of MSP2N2. Tritiated flunitrazepam ([^3^H]-flunitrazepam; PerkinElmer) was used as the radioligand, while 0.4 mM clorazepate (MilliporeSigma; 2× background solution) was used to determine nonspecific binding. For the saturation binding assay, cerebellar GABA_A_Rs were first mixed with SPA beads (2× bead solution), while serial dilutions were prepared for the radioligand (2× ligand solution) ranging from 1 nM to 80 nM. For the competition assay, cerebellar GABA_A_Rs were first mixed with SPA beads in the presence of 16 nM radioligand (2× bead solution), while serial dilutions were prepared for the PZ-II-029 (2× ligand solution) ranging from 0.2 nM to 3.2 μM. Equal volumes of the 2× bead solution were then combined with either the 2× ligand solution (in triplicate) or the 2× background solution in a 96-well plate. The final assay conditions were 0.5 mg/mL SPA beads, ~0.3 nM native receptors. Following a 3-h incubation, the plate was read using a MicroBeta TriLux scintillation counter. Data analysis was performed in Python using SciPy, applying either a one-site binding or competitive inhibition model.

### Cryo-EM Sample Preparation.

Gold Quantifoil grids (200 mesh, either 2/1 or 2/2) overlaid with a 2-nm continuous carbon layer were glow discharged in amylamine at 15 mA for 30 s. The inclusion of fluorinated detergents, either fluorinated phosphocholine-8 (FOC) or fluorinated octyl maltoside (FOM) (44), was found to be essential for achieving uniform ice formation on these grids. FOM was prepared at a final concentration of 10 mM in TBS buffer either by itself or with 600 μM PZ-II-029 and 5% DMSO. Then, 0.5 μL aliquots were dispensed into PCR tubes in advance. For vitrification, 5 μL of purified cerebellar receptor in nanodiscs was added to the PCR tube and rapidly mixed by pipetting. Within 10 s, 2.5 μL of the mixture was applied to the grid and incubated for 30 s. The grid was subsequently blotted using a Mark IV Vitrobot under conditions of 100% humidity and 16 °C, followed by plunge-freezing in an ethane-propane mixture.

### Cryo-EM Data Acquisition.

The GABA dataset was acquired using a 300-keV Titan Krios equipped with a BioQuantum energy filter at the Janelia cryo-EM facility. Data collection was automated via SerialEM, following the parameters and workflow outlined below: Defoci were set within the range of -0.9 to -2.5 μm; holes with appropriate ice thickness were identified and selected using the built-in hole finder module; these selected holes were then combined to generate multishot acquisition targets. These movies were acquired using a K3 direct electron detector under the correlated double sampling (CDS) mode. A total electron dose of 43 electron/Å^2^ was fractionated into 40 frames with a dose rate of 7 electron/(pixel * second). The PZ-II-029/GABA dataset was collected on a 300-keV Titan Krios equipped with a Selectris X energy filter at the Stanford-SLAC cryo-EM center. Fringe-free multishot-multihole data acquisition was automated using EPU, with defocus ranging from −0.8 to −2.0 μm. Half of the dataset was collected with the stage tilted at 30°. These movies were captured with a Falcon 4i camera, saved into bin1 TIFF files that were fractionated into 40 frames. Each movie has a total dose of roughly 45 electron/Å^2^, with a dose rate of 8 electron/(pixel * second).

### Cryo-EM Data Processing.

Cryo-EM movies were imported into CryoSPARC (45) v 4.6.2 and subjected to motion correction using the patch motion correction integrated within CryoSPARC. Subsequently, contrast transfer function (CTF) parameters were calculated using the built-in patch CTF estimation tool. For each dataset, 2D class averages of particles, initially picked using glob-picker from approximately 1,000 micrographs, were employed as templates to perform particle picking across all micrographs. GABA_A_R particles were separated from junk particles using 2D classification, and *ab initio* models were generated for both the GABA_A_R and the junk decoys, respectively. Using these 3D models, multiple rounds of heterogeneous refinement were performed on all extracted bin2 particles to isolate GABA_A_R particles. The cleaned GABA_A_R particles then underwent multiple rounds of 2D classification to further select those of the highest quality. A nonuniform refinement (46) (NU-refinement) was carried out to align these particles to a consensus 3D model, which was necessary for downstream focused classification to separate different receptor assemblies.

Two stages of focused 3D classification without alignment were performed in CryoSPARC to elucidate the receptor assembly. The first classification separated particles into groups based on the number of bound Fab fragments, while the second classification further distinguished receptor assemblies within each group. The focus mask and classification parameters were extensively tested. Ultimately, we selected a mask covering the complete ECD of the receptor along with the bound Fabs and a filtering resolution of 5 Å for the first step. Two-Fab classes and one-Fab classes were separately refined using NU-refinement. We then used a mask covering the ECD of the receptor and a filtering resolution of 4 Å for the second classification. All classes were further refined using NU-refinement.

To interpret the subunit for each 3D class, visual inspection of the glycosylation was used to identify α, β, and γ subunits. Automatic model building with ModelAngelo (47) was then run with unsharpened 3D volumes and protein sequences of rat GABA_A_R subunits identified from mass spectrometry. For a subunit to be identified as a particular subunit, more than 50% of the ECD residues need to be modeled by ModelAngelo, which corresponds to roughly 100 amino acids. In cases where the modeled ECD consists of more than one subunit, the secondary subunit needs to make up 25% of the ECD residues, which is roughly 50 residues. The counting process of modeled ECD residues for individual subunits was automated using a Python script invoked within ChimeraX (48). Only cryo-EM maps with all five subunits identified were manually inspected and used for defining receptor assemblies. The Fourier Shell Correlation (FSC) curves for cryo-EM reconstructions were from the output of NU-refinement jobs. The local resolution estimation was carried out in CryoSPARC. The plots of angular distribution of particles were prepared using pyem (49).

### Model Building.

Cryo-EM maps were sharpened with EMReady (50) for visualization, model building, and refinement purposes. Native mouse GABA_A_R model 8FOI was used as the starting point for model building. For subunits identified in this study, molecular models of rat α6, β1, and β3 were predicted using the AlphaFold3 server (51), and residues were renumbered to reflect the removal of signal peptides. Some receptor assemblies have ambiguity in subunit assignment, containing primary and secondary subunits at a specific position. In these cases, only a single model with the primary subunit was constructed. All molecular models were then fitted into cryo-EM maps using the fit_in_map function of chimeraX and imported to coot (52) for structure editing, including addition of *N*-glycosylation, flexible fitting, and removal of residues that were not supported by the experimental data. In particular, the transmembrane helices were removed due to structural heterogeneity in the reconstructed cryo-EM maps. To model the compound PZ-II-029 into the cryo-EM maps, phenix.elbow (53) was used to optimize its geometry and generate the structural constraints for flexible fitting in coot. After obtaining the initial modeling, multiple rounds of phenix.real_space_refinement (54) and editing in coot were carried out to improve the model quality. Libplot+ (55) was used to visualize the ligand protein interactions.

## Supplementary Material

Appendix 01 (PDF)

## Data Availability

All atomic models and cryo-EM maps of cerebellar GABA_A_Rs have been deposited in the Protein Data Bank (PDB) and Electron Microscopy Data Bank (EMDB): β1-α1-β2-α1-γ2 in complex with GABA (PDB 9OUQ, EMD-70876), β2-α1-β2-α1-γ2 in complex with GABA (PDB 9OUR, EMD-70877), β2-α1-β1-α6-γ2 in complex with GABA (PDB 9OUP, EMD-70875), β1-α1-β1-α1-γ2 in complex with GABA and PZ-II-029 (PDB 9OUO, EMD-70874), β1-α1-β2-α1-γ2 in complex with GABA and PZ-II-029 (PDB 9OV4, EMD-70889), β2-α1-β2-α1-γ2 in complex with GABA and PZ-II-029 (PDB 9OUN, EMD-70873), and β2-α1-β1-α6-γ2 in complex with PZ-II-029/GABA (PDB 9OUM, EMD-70872). The mass spectrometry data of rat cerebellar GABA_A_R sample and the python script used for counting modeled residues have been uploaded to Zenodo (56, 57). Immunofluorescence data have been deposited in the BioImage Archive (S-BIAD2097).
