# Supplementary material for: Molecular assemblies and pharmacology of cerebellar GABAA receptors
Source: Proc Natl Acad Sci U S A. 2026 Feb 6;123(6):e2524504123. doi: 10.1073/pnas.2524504123 (PMC12890884; doi:10.1073/pnas.2524504123)
Supplement: Supplementary file 1 — Appendix 01 (PDF) [file pnas.2524504123.sapp.pdf]

PNAS

Supporting Information for

Molecular assemblies and pharmacology of cerebellar $GABA_A$ receptors

Chang Sun[1,‡], Jennifer N. Jahncke[1], Kevin M. Wright[1], Eric Gouaux[1,2,*]

[1]Vollum Institute, Oregon Health and Science University, 3232 SW Research Drive, Portland, Oregon 97239.

[2]Howard Hughes Medical Institute, Oregon Health and Science University, 3232 SW Research Drive, Portland, Oregon 97239.

‡Present address: Department of Structural Biology, Van Andel Institute, 333 Bostwick Ave. NE, Grand Rapids, Michigan 49503

[*]Correspondence to Eric Gouaux

Email: gouauxe@ohsu.edu

This PDF file includes:

Supplementary Figures S1 to S12
Supplementary Tables S1 to S2

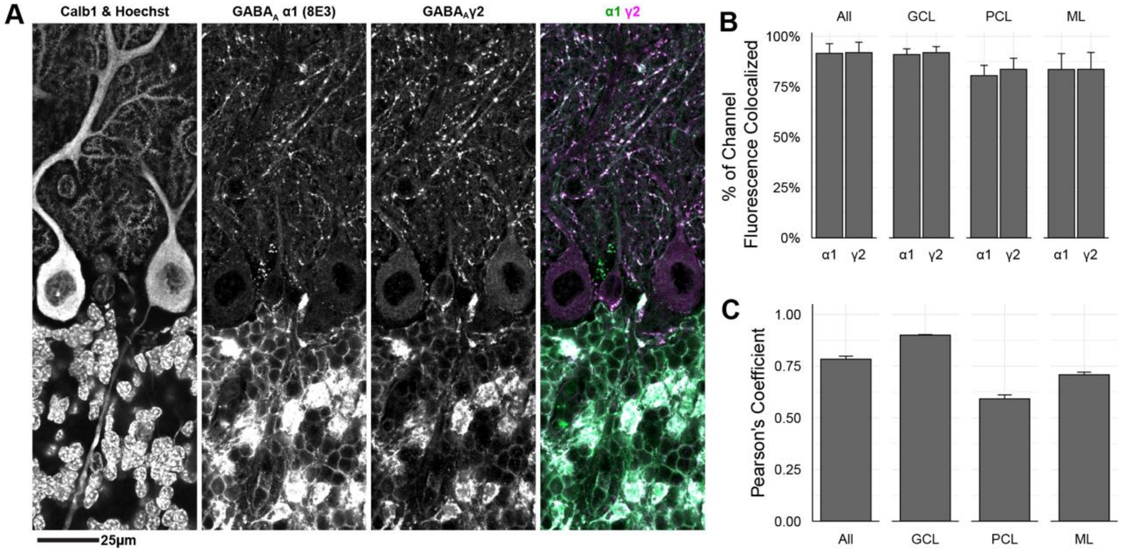

Figure S1. Colocalization of GABA$_A$R α1 and γ2 subunits in the rat cerebellar cortex. (**A**) Representative confocal images showing the distribution of the GABA$_A$ receptor (GABA$_A$R) α1 subunit (labeled with the monoclonal 8E3 antibody) and γ2 subunit (labeled with a commercial polyclonal antibody) across the cerebellar cortex. Layers of the cerebellar cortex including the granule cell layer (GCL), Purkinje cell layer (PCL), and molecular layer (ML) are visible. (**B**) Quantification of the percentage of colocalized fluorescence between α1 and γ2 subunits, shown for the entire image volume ("all") and individual layer compartments (GCL, PCL, ML). Thresholds to separate signal and background were automatically determined using Imaris**.** Error bars represent standard error of the mean (n = 4). (**C**) Pearson's correlation coefficients for α1 and γ2 subunit colocalization in the same regions as in (B), indicating strong colocalization overall, particularly within the granule cell layer. Error bars represent standard error of the mean (n = 4).

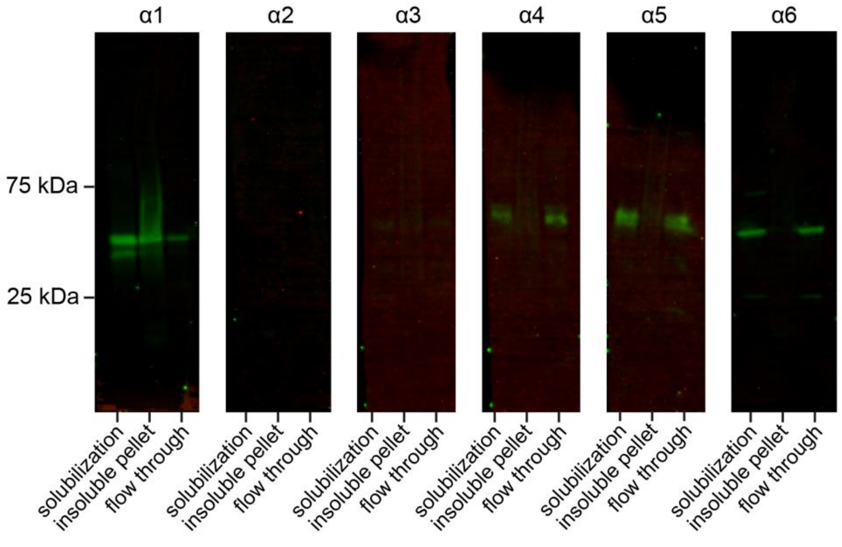

Figure S2. Western blot analysis of the GABA$_A$R α subunits in the biochemical purification. In each immunoblot, lanes from left to right are solubilized membrane fraction, resuspended insoluble pellet, and the flowthrough after the affinity capture by the streptactin-XT resin.

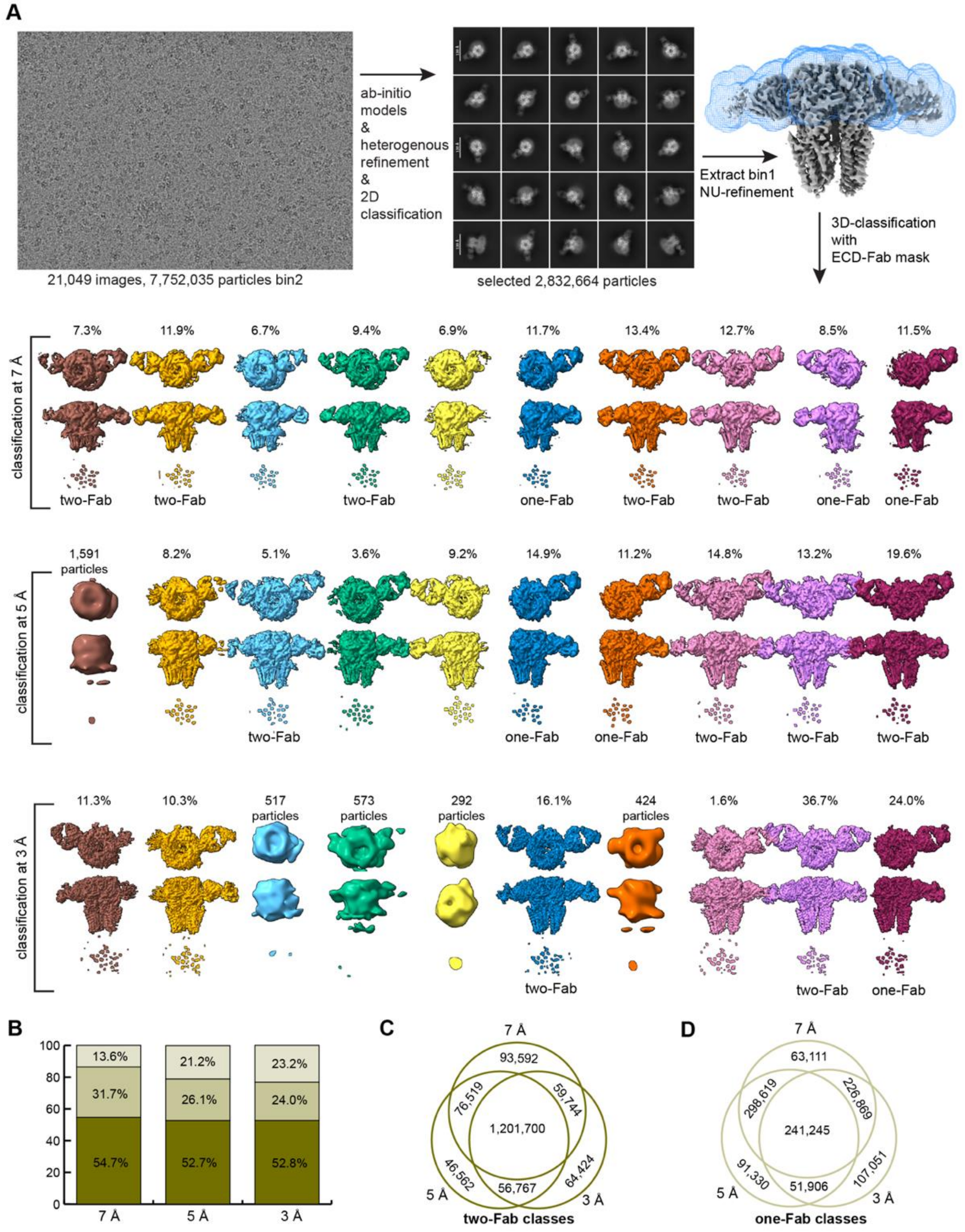

Figure S3. **Cryo-EM data processing of the GABA dataset to separate one-Fab receptors from two-Fab receptors.** (**A**) Workflow of cryo-EM data processing. Each 3D class is shown with a top-down, side, and transmembrane slice view at a threshold of 6 standard deviation of the map. (**B**) Particle distribution of unclear classes (light green), one-Fab classes (green), and two-Fab classes (dark green) from 3D classification using different resolution cutoffs. (**C**) Venn diagram showing particle overlaps among two-Fab classes from 3D classification at different resolution cutoffs. (**D**) Venn diagram showing particle overlaps among one-Fab classes from 3D classification at different resolution cutoffs.

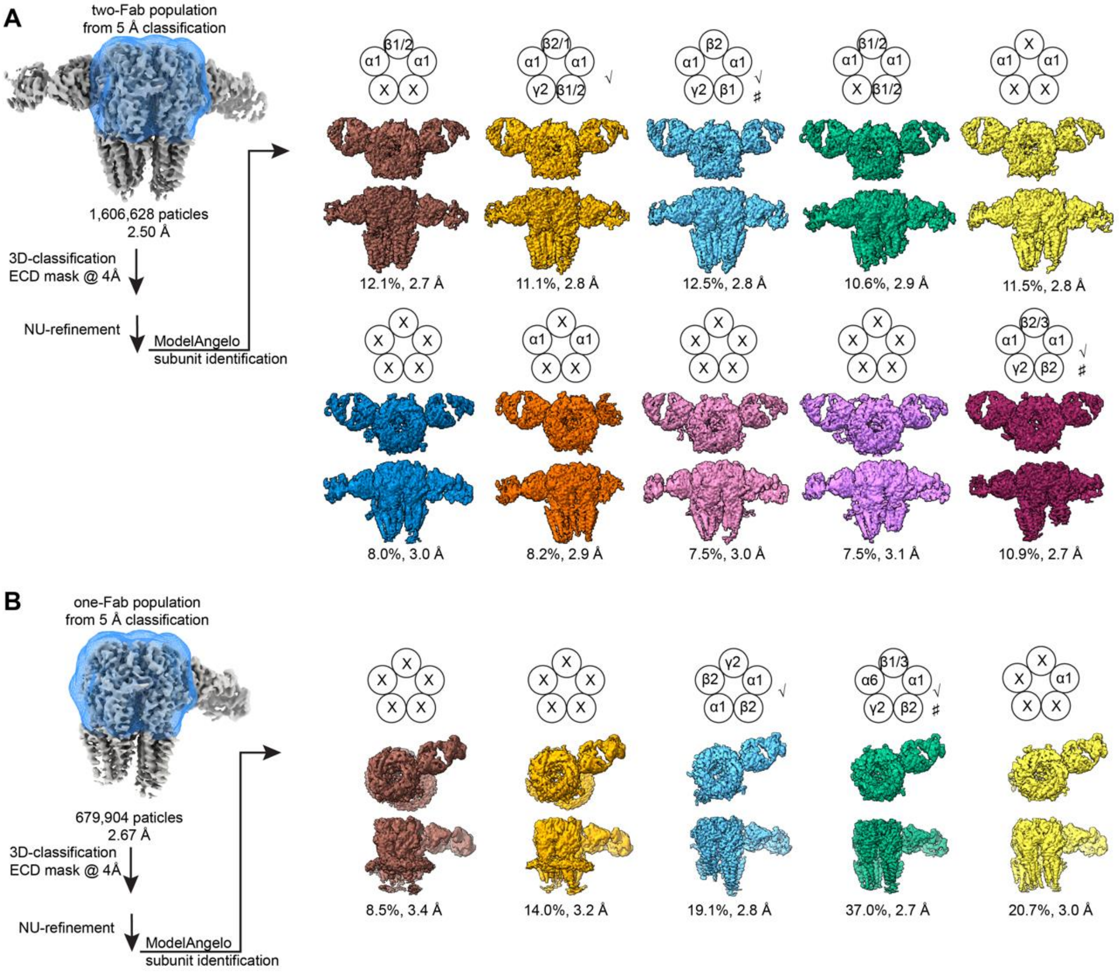

Figure S4. Cryo-EM data processing of the GABA dataset to identify receptor assemblies for the two-Fab receptors (A) and the one-Fab receptors (B). In each case, 3D classification was carried out with a focus mask covering the extracellular domain (ECD) of the receptor. All classes were further refined using NU-refinement, and the unsharpened maps were used for subunit identification in ModelAngelo using protein sequences of rat GABA$_A$R subunits identified from mass spectrometry. In the schematics for receptor assembly, "X" denotes that a single subunit with sufficient ECD residues (more than 50%) cannot be modeled at this position by ModelAngelo. If all five subunits can be identified for a cryo-EM class using ModelAngelo, a "√" symbol is displayed next to the receptor assembly schematic. Additionally, a "#" symbol indicates that this receptor assembly underwent further analysis through model building and refinement.

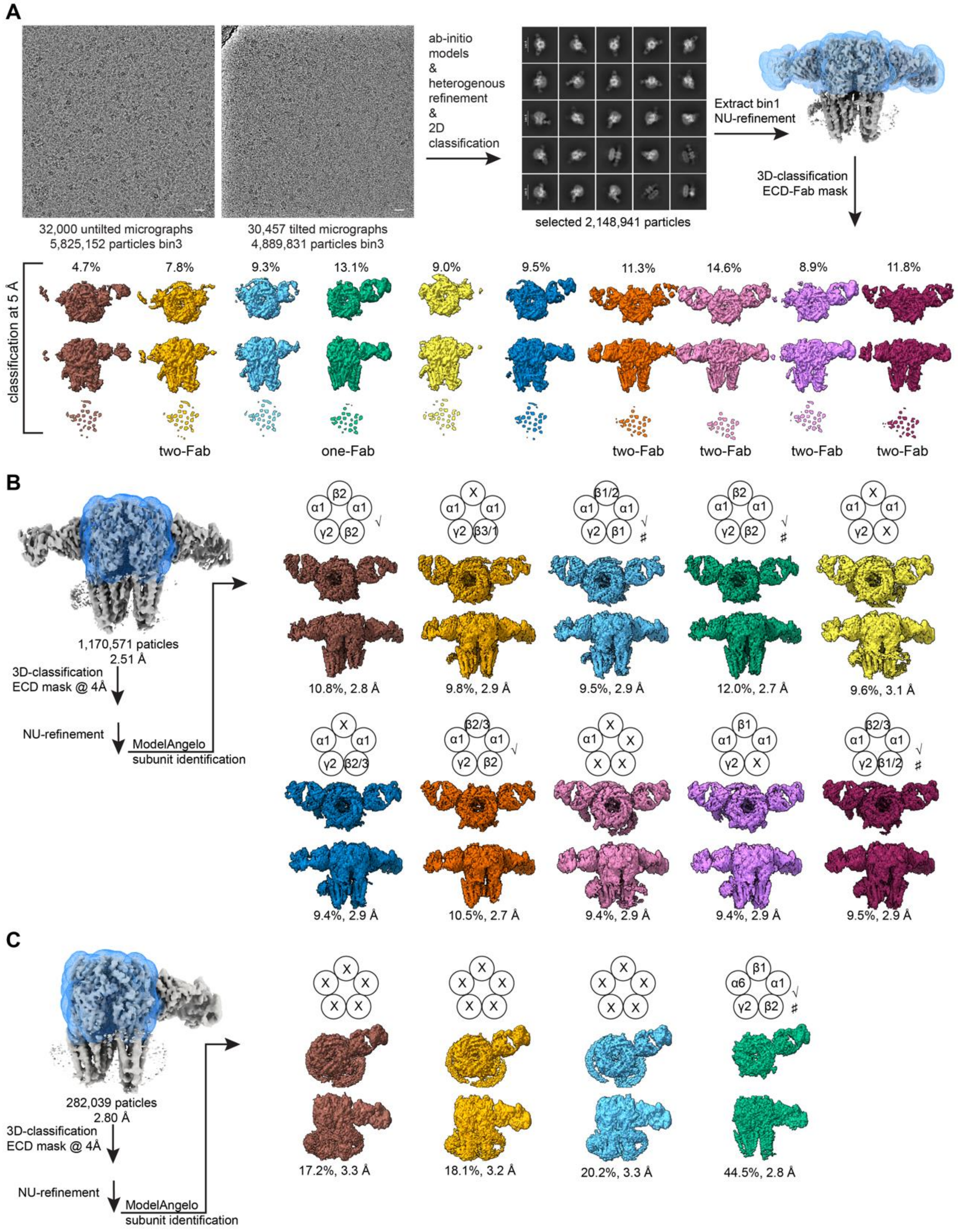

Figure S5. Cryo-EM data processing of the PZ-II-029/GABA dataset. (**A**) Cryo-EM data processing to separate one-Fab receptors from two-Fab receptors. (**B**) Cryo-EM data processing to identify receptor assemblies within the two-Fab population. (**C**) Cryo-EM data processing to identify receptor assemblies within the one-Fab population.

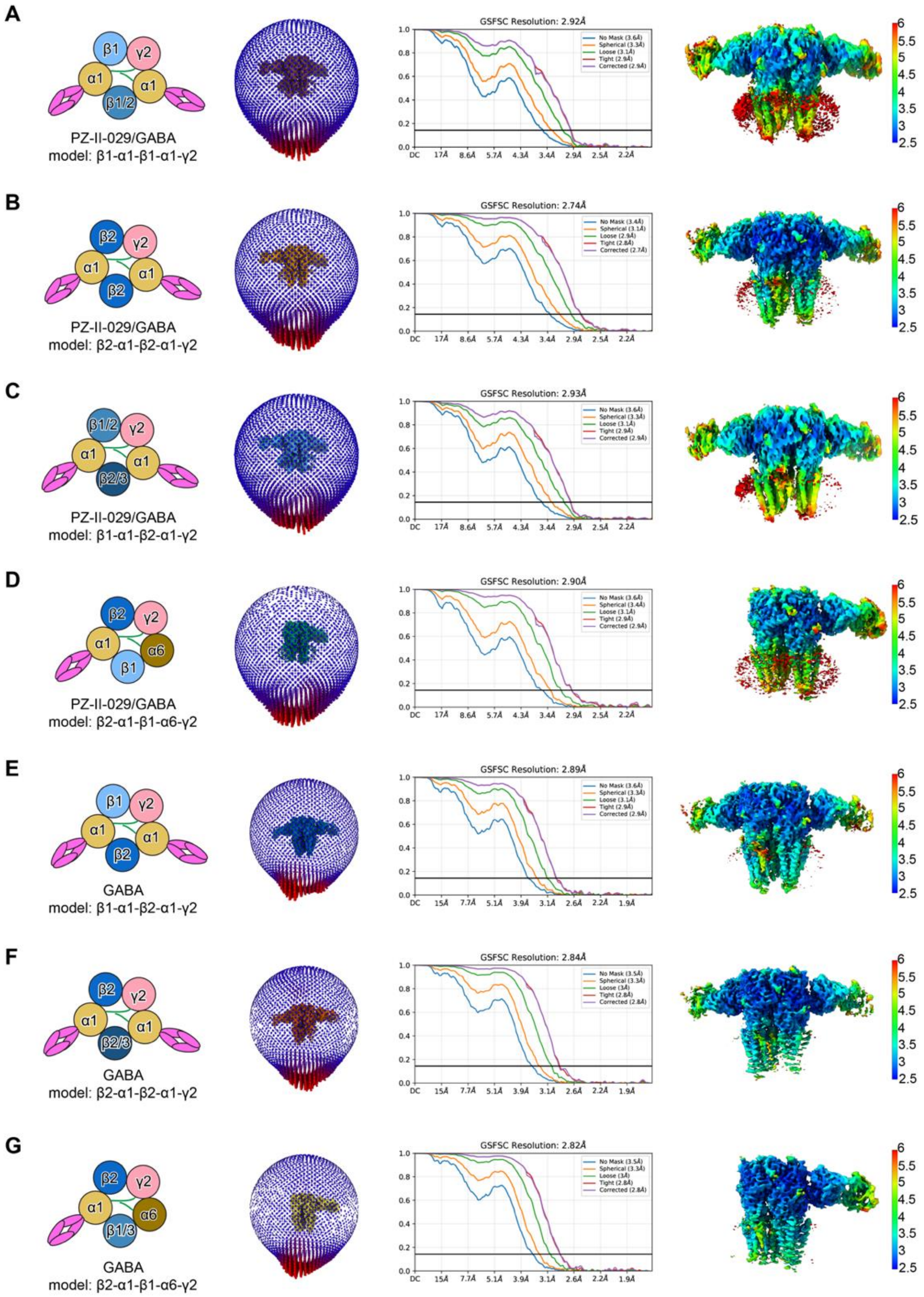

Figure S6. Statistics of final cryo-EM reconstructions. (**A-D**) Angular distribution, Fourier Shell Correlation (FSC) curve, and local resolution estimation for reconstruction from the PZ-II-029/GABA dataset. (**E-G**) Angular distribution, FSC curve, and local resolution estimation for reconstruction from the GABA dataset.

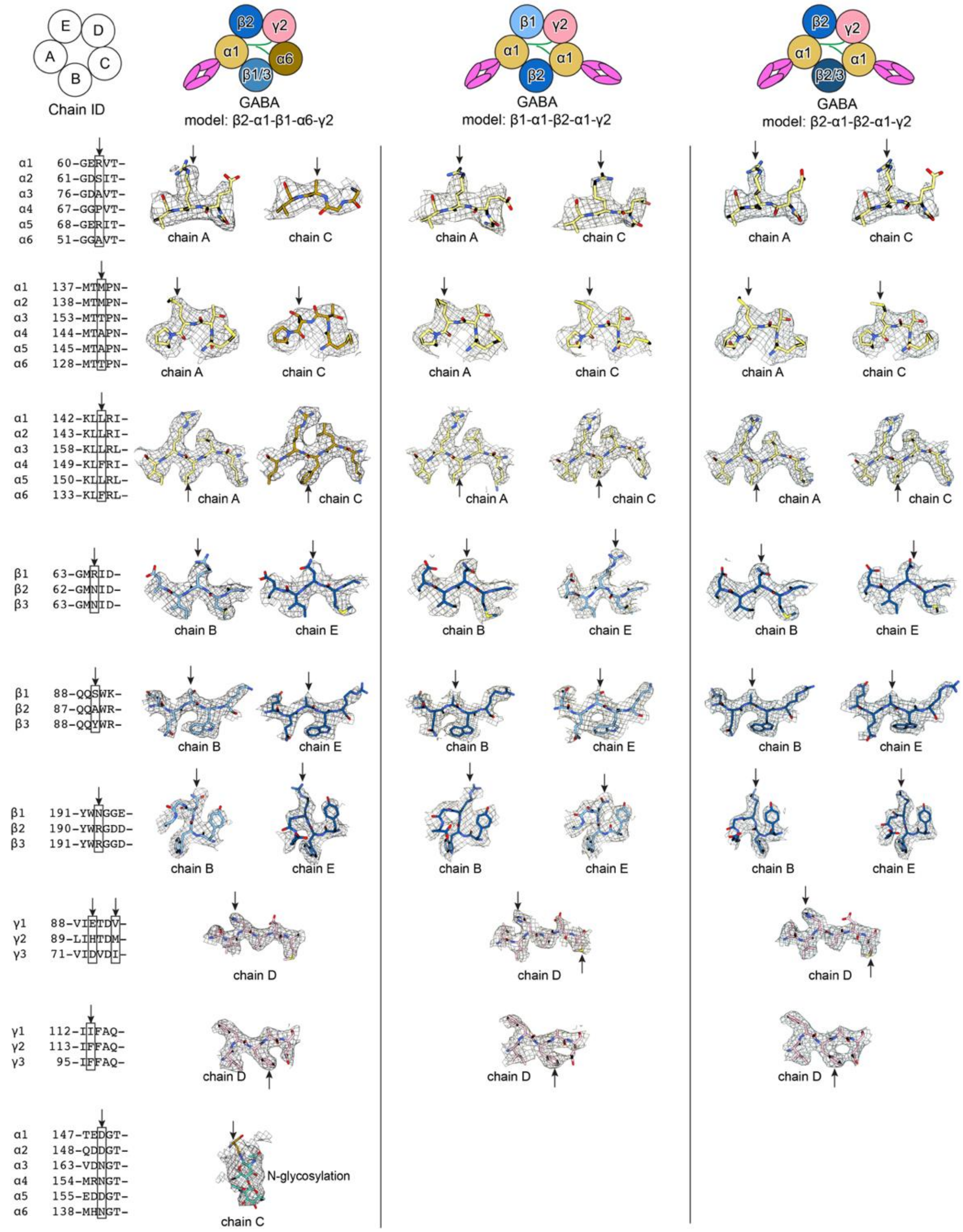

Figure S7. Inspection of cryo-EM density for subunit assignment. The figure's top section displays schematics of the three identified and modeled receptor assemblies from the GABA dataset, with the five $GABA_AR$ subunits labeled by chain IDs and color-coded. Aligned sequence segments are displayed on the left of each row, with distinguishing residues enclosed in boxes. Cryo-EM densities contoured at the same level from these three populations (separated by a vertical bar) are shown, with arrows pointing to the distinguishing residues. The final row highlights the *N*-glycosylation present in the α6 subunit, which is absent in the α1, α2, and α5 subunits.

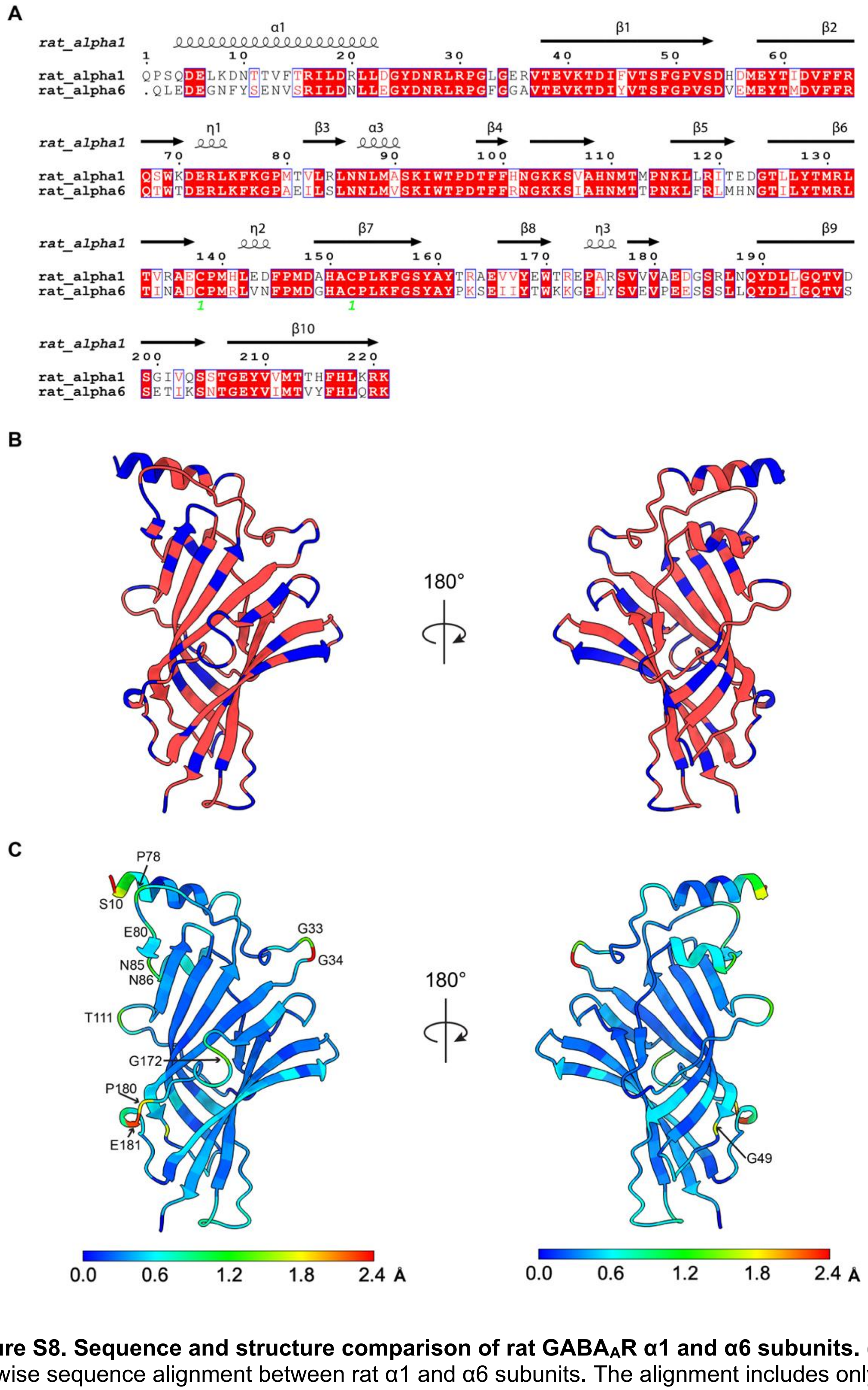

Figure S8. Sequence and structure comparison of rat GABA$_A$R α1 and α6 subunits. (**A**) pairwise sequence alignment between rat α1 and α6 subunits. The alignment includes only the mature ECD of both subunits for clarity. Secondary structure elements, including the ten prominent β strands, are indicated above the sequences. (**B**) Cartoon representation of the rat α6

subunit colored by residue identity compared to rat α1 based on a pairwise sequence alignment, with conserved residues colored in red. (**C**) Cartoon representation of the rat α6 subunit colored by the per-residue Cα distance after structural superposition with rat α1. Residues displaying significant Cα displacements (>1 Å) are labeled.

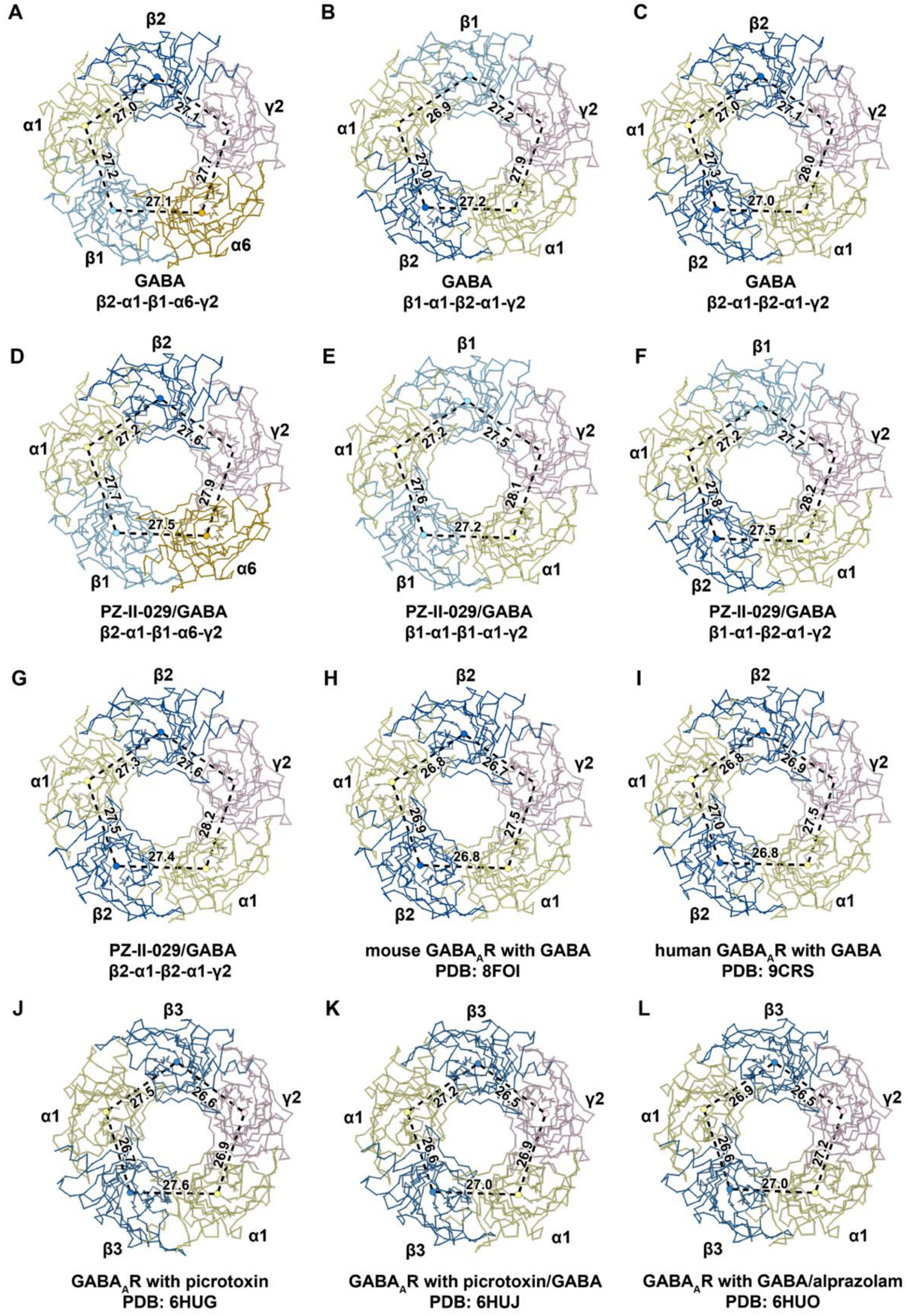
A
β2
α1
γ2
β1
α6
27.0
27.1
27.2
27.7
27.1
GABA
β2-α1-β1-α6-γ2
B
β1
α1
γ2
β2
α1
26.9
27.2
27.0
27.9
27.2
GABA
β1-α1-β2-α1-γ2
C
β2
α1
γ2
β2
α1
27.0
27.1
27.3
28.0
27.0
GABA
β2-α1-β2-α1-γ2
D
β2
α1
γ2
β1
α6
27.2
27.6
27.7
27.9
27.5
PZ-II-029/GABA
β2-α1-β1-α6-γ2
E
β1
α1
γ2
β1
α1
27.2
27.5
27.6
28.1
27.2
PZ-II-029/GABA
β1-α1-β1-α1-γ2
F
β1
α1
γ2
β2
α1
27.2
27.7
27.8
28.2
27.5
PZ-II-029/GABA
β1-α1-β2-α1-γ2
G
β2
α1
γ2
β2
α1
27.3
27.6
27.5
28.2
27.4
PZ-II-029/GABA
β2-α1-β2-α1-γ2
H
β2
α1
γ2
β2
α1
26.8
26.7
26.9
27.5
26.8
mouse GABAAR with GABA
PDB: 8FOI
I
β2
α1
γ2
β2
α1
26.8
26.9
27.0
27.5
26.8
human GABAAR with GABA
PDB: 9CRS
J
β3
α1
γ2
β3
α1
27.5
26.6
26.7
26.9
27.6
GABAAR with picrotoxin
PDB: 6HUG
K
β3
α1
γ2
β3
α1
27.2
26.5
26.6
26.9
27.0
GABAAR with picrotoxin/GABA
PDB: 6HUJ
L
β3
α1
γ2
β3
α1
26.9
26.5
26.6
27.2
27.0
GABAAR with GABA/alprazolam
PDB: 6HUO

Figure S9. Analysis of the extracellular domain geometry of native $GABA_A$Rs from the GABA dataset (**A**) β2-α1-β1-α6-γ2, (**B**) β1-α1-β2-α1-γ2, (**C**) β2-α1-β2-α1-γ2, from the PZ-II-029/GABA dataset (**D**) β2-α1-β1-α6-γ2, (**E**) β1-α1-β1-α1-γ2, (**F**) β1-α1-β2-α1-γ2, (**G**) β2-α1-β2-α1-γ2, and from previous studies (**H**) β2-α1-β2-α1-γ2 (PDB: 8FOI, mouse $GABA_A$R in complex with GABA and allopregnanolone), (**I**) β2-α1-β2-α1-γ2 (PDB: 9CRS, human $GABA_A$R in complex with GABA), (**J**) β3-α1-β3-α1-γ2 (PDB: 6HUG, recombinant human $GABA_A$R in complex with picrotoxin), (**K**) β3-α1-β3-α1-γ2 (PDB: 6HUJ, recombinant human $GABA_A$R in complex with picrotoxin and GABA), (**L**) β3-α1-β3-α1-γ2 (PDB: 6HUG, recombinant human $GABA_A$R in complex with GABA and alprazolam). In each panel, the Cα trace and center of mass of the individual ECD are shown. The distances between center of masses are measured (in Å) and shown as well. The binding of GABA at the β^+/α^- interface closes the loop C and reduces the distance between β and α subunits, while the binding of alprazolam and PZ-II-029 at the α^+/γ^- interface increases the distance between α and γ subunits.

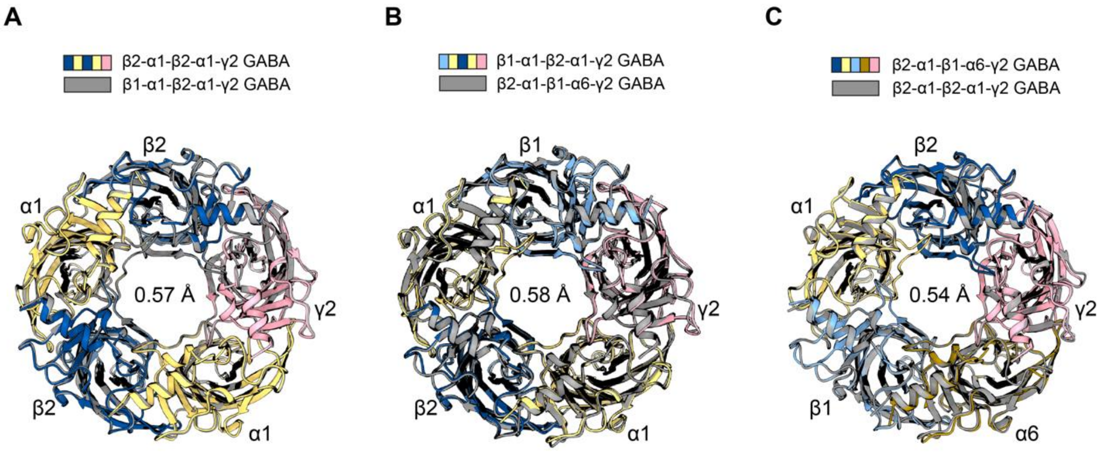

A

	β2	α1	β2	α1	γ2
β1	0.45 Å		0.50 Å		
α1		0.46 Å		0.45 Å	
β2	0.52 Å		0.45 Å		
α1		0.48 Å		0.39 Å	
γ2					0.43 Å

B

	β1	α1	β2	α1	γ2
β2	0.42 Å		0.47 Å		
α1		0.35 Å		0.44 Å	
β1	0.52 Å		0.44 Å		
α6		0.76 Å		0.71 Å	
γ2					0.52 Å

C

	β2	α1	β1	α6	γ2
β2	0.24 Å		0.42 Å		
α1		0.32 Å		0.65 Å	
β2	0.27 Å		0.32 Å		
α1		0.29 Å		0.63 Å	
γ2					0.55 Å

Figure S10. Structural comparison of cerebellar GABA$_A$R assemblies from the GABA dataset (**A**) between β2-α1-β2-α1-γ2 (colored) and β1-α1-β2-α1-γ2 (gray), (**B**) between β1-α1-β2-α1-γ2 (colored) and β2-α1-β1-α6-γ2 (gray), (**C**) between β2-α1-β1-α6-γ2 (colored) and β2-α1-β2-α1-γ2 (gray). In each panel, the two structures were superimposed based on either the full ECD or individual subunits. The top-down view depicts the full ECD-aligned overlay, and the root-mean-square deviation (RMSD) of Cα atoms between the assemblies is indicated at the center. The lower table shows pairwise RMSD matrices between corresponding subunits (α, β, and γ) from the two structures, highlighting conformational differences that are dependent on subunit and position in the assembly.

A

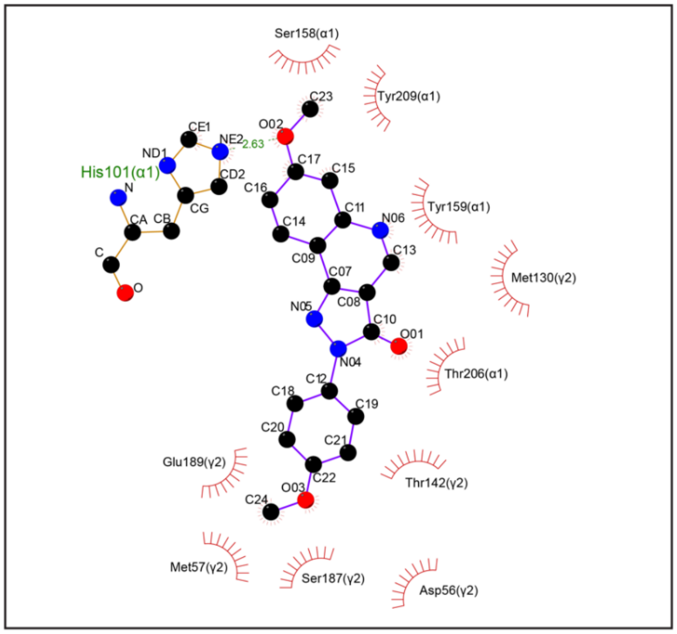

β2-α1-β2-α1-γ2

B

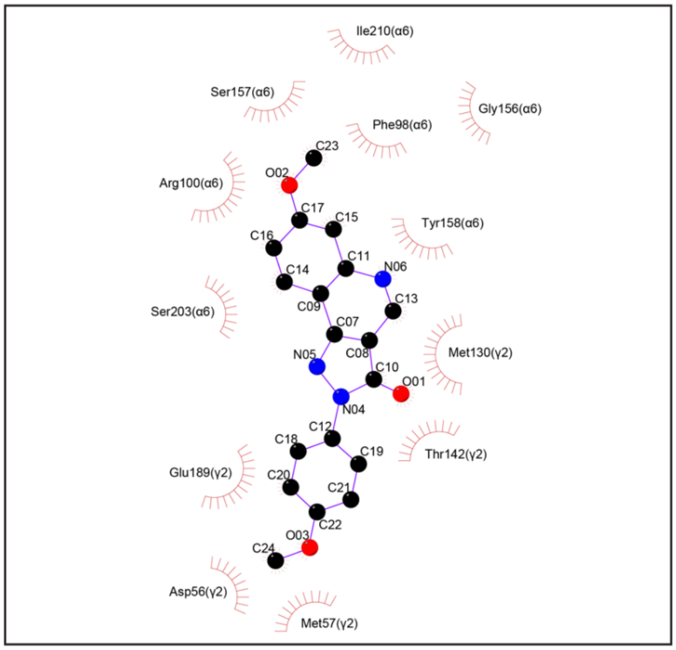

β2-α1-β1-α6-γ2

Figure S11. Diagram of ligand-protein interactions between PZ-II-029 and native $GABA_A$Rs at the $\alpha1^+/\gamma2^-$ pocket (A) and the $\alpha6^+/\gamma2^-$ pocket (B).

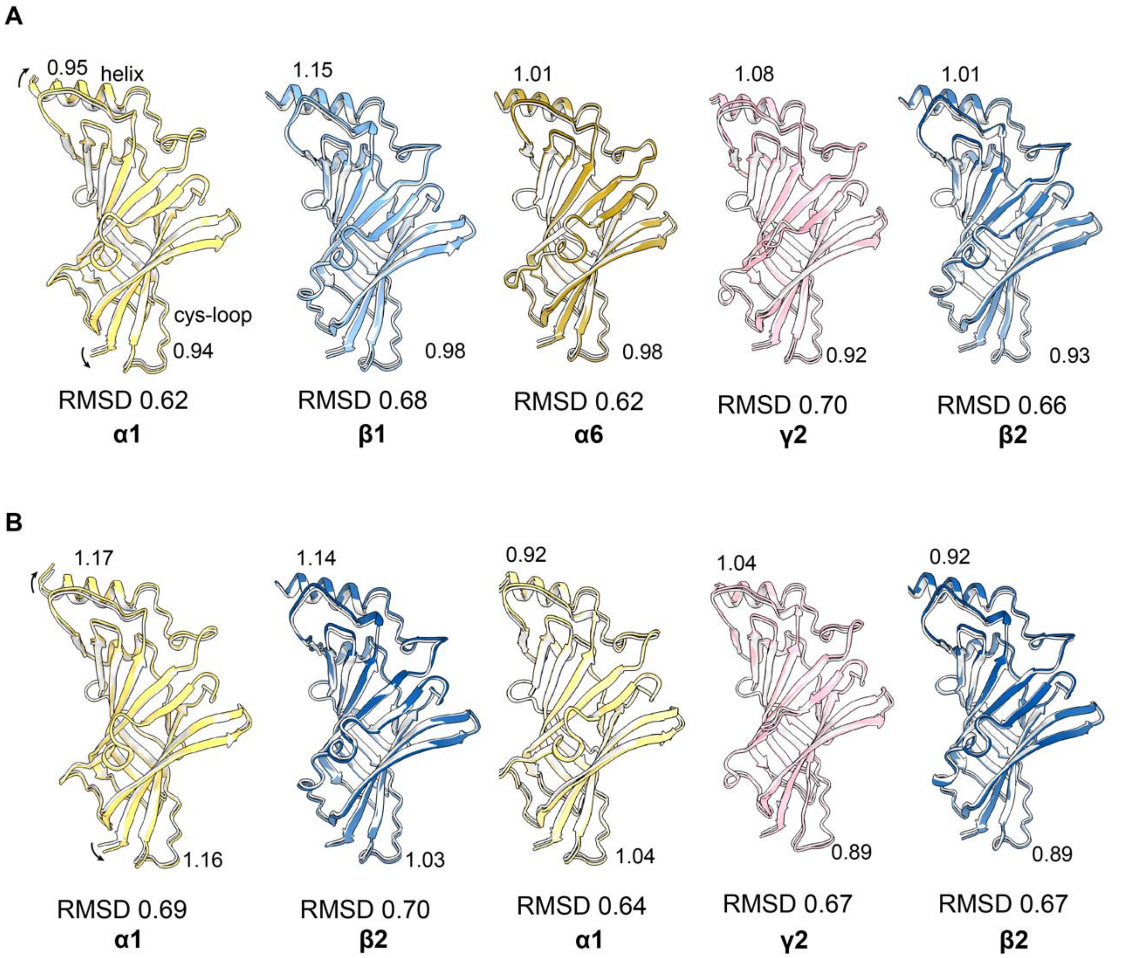

Figure S12. Structural alignments of individual subunits of (A) the β2-α1-β1-α6-γ2 or (B) the β2-α1-β2-α1-γ2 receptor with (colored) or without (white) ligand PZ-II-029. In all cases, the alignment was carried out with all the Cα atoms of the ECD of the subunit inspected. The RMSDs (in Å) of the Cα atoms of the complete ECD, the N-terminal helix, and the cys-loop were measured and displayed. Upon binding of PZ-II-029, individual ECD rearranges to exhibit a more extended configuration along the channel pore axis.

Table S1. Cryo-EM data collection.

	Native GABA$_A$R + GABA	Native GABA$_A$R + GABA + PZ-II-029
**Data collection and processing**		
Microscope	PNCC Krios	S2C2 Krios
Electron Gun	XFEG	XFEG
Voltage (kV)	300	300
Energy filter slit width (eV)	20	10
Detector	K3	Falcon 4i
Operation mode	CDS	
Flux on detector (e^-/pix/sec)	7.7	8
Total electron exposure on sample (e^-/Å^2)	43	45
Number of movie frames	40	40
Magnification	81K	130K
Pixel size (Å)	0.857	0.956
Targeted defocus range (μm)	1.2–2.5	0.8–2.0
Number of collected movies	21,049	62,457
Symmetry imposed	C1	C1
Initial particle images (no.)	7,752,035	10,714,983
GABA$_A$R particle images (no.)	2,832,664	2,148,941
Final particle images for modeling(no.)	201,371 (β1-α1-β2-α1-γ2)<br>174,593 (β2-α1-β2-α1-γ2)<br>251,382 (β2-α1-β1-α6-γ2)	126,564 (β1-α1-β1-α1-γ2)<br>126,837 (β1-α1-β2-α1-γ2)<br>159,009 (β2-α1-β2-α1-γ2)<br>125,387 (β2-α1-β1-α6-γ2)
Map resolution (Å)<br>FSC=0.143	2.89 Å (β1-α1-β2-α1-γ2)<br>2.84 Å (β2-α1-β2-α1-γ2)<br>2.82 Å (β2-α1-β1-α6-γ2)	2.92 Å (β1-α1-β1-α1-γ2)<br>2.93 Å (β1-α1-β2-α1-γ2)<br>2.74 Å (β2-α1-β2-α1-γ2)<br>2.90 Å (β2-α1-β1-α6-γ2)
EMPIAR code		

Table S2. Model refinement and validation statistics.

	(β1-α1-β2-α1-γ2) (GABA)	(β2-α1-β2-α1-γ2) (GABA)	(β2-α1-β1-α6-γ2) (GABA)	(β1-α1-β1-α1-γ2) (GABA +PZ-II-029)	(β1-α1-β2-α1-γ2) (GABA +PZ-II-029)	(β2-α1-β2-α1-γ2) (GABA +PZ-II-029)	(β2-α1-β1-α6-γ2) (GABA +PZ-II-029)
**EMDB ID**	EMD-70876	EMD-70877	EMD-70875	EMD-70874	EMD-70889	EMD-70873	EMD-70872
**PDB ID**	9OUQ	9OUR	9OUP	9OUO	9OV4	9OUN	9OUM
Model resolution (Å) FSC=0.143	2.89	2.84	2.82	2.92	2.93	2.74	2.90
Map sharpening *B* factor (Å^2)	0	0	0	0	0	0	0
**Model composition**							
Non-hydrogen atoms	12,351	12,361	10,643	12,337	12,369	12,385	10,661
Protein residues	1,498	1,499	1,277	1,497	1,498	1,499	1,277
Glycan molecules	25	25	25	23	25	25	25
Ligand molecules	2	2	2	3	3	3	3
***B* factors (Å^2)**							
Protein	70	46	51	59	67	46	50
Ligands	92	75	89	92	114	73	87
**R.m.s. deviations**							
Bond lengths (Å)	0.006	0.005	0.004	0.005	0.003	0.004	0.003
Bond angles (°)	0.684	0.655	0.612	0.658	0.557	0.609	0.578
**Validation**							
MolProbity score	2.44	2.09	1.89	2.20	1.85	1.78	1.86
Clashscore	10.06	7.22	6.37	7.04	5.34	3.69	4.37
Poor rotamers (%)	5.07	2.64	2.54	3.42	2.20	2.34	3.33
**Ramachandran plot**							
Favored (%)	94.39	94.73	96.44	93.91	95.68	95.07	96.28
Allowed (%)	5.61	5.29	3.48	6.02	4.32	4.86	3.72
Disallowed (%)	0.00	0.07	0.08	0.07	0.00	0.07	0.00
